# Clinicopathological Characteristics and Breast Cancer–Specific Survival of Patients With Single Hormone Receptor–Positive Breast Cancer

**DOI:** 10.1001/jamanetworkopen.2019.18160

**Published:** 2020-01-03

**Authors:** Yunhai Li, Dejuan Yang, Xuedong Yin, Xiang Zhang, Jiefeng Huang, Yusheng Wu, Mengxue Wang, Zhiying Yi, Hongyuan Li, Hongzhong Li, Guosheng Ren

**Affiliations:** 1 Department of Endocrine and Breast Surgery, First Affiliated Hospital of Chongqing Medical University, Chongqing, China; 2 Chongqing Key Laboratory of Molecular Oncology and Epigenetics, First Affiliated Hospital of Chongqing Medical University, Chongqing, China

## Abstract

**Importance:**

Steroid hormone receptors, including estrogen receptor (ER) and progesterone receptor (PR), are crucial biomarkers in breast cancer (BC). However, limited data are available regarding single hormone receptor–positive (ER-positive/PR-negative and ER-negative/PR-positive) subtypes, rendering treatment decision and survival forecast difficult in patients with these BC subtypes.

**Objective:**

To investigate the clinicopathological characteristics and BC-specific survival (BCSS) of patients with single hormone receptor–positive BC.

**Design, Setting, and Participants:**

In this cohort study, data on patients diagnosed with BC between 1990 and 2015 were retrieved from the Surveillance, Epidemiology, and End Results database (N = 1 158 032). Patients lacking information on ER status, PR status, or BCSS were excluded (n = 334 633). Comparisons were performed between single hormone receptor–positive BC and double hormone receptor–positive/double hormone receptor–negative BC. The dates of analysis were September 1, 2018, to June 31, 2019.

**Main Outcomes and Measures:**

The BCSS of patients with single hormone receptor–positive BC.

**Results:**

This cohort study included 823 399 patients with BC (818 002 women and 5397 men). The median (range) age at diagnosis was 60 (8-108) years, and the median (range) follow-up duration was 71 (0-311) months. The percentages of ER-positive/PR-positive, ER-positive/PR-negative, ER-negative/PR-positive, and ER-negative/PR-negative cases were 67.2%, 12.2%, 1.6%, and 19.0%, respectively. Single hormone receptor–positive subtypes showed distinct clinical characteristics compared with double hormone receptor–positive/double hormone receptor–negative subtypes. Multivariable Cox proportional hazards regression analysis showed that patients with ER-positive/PR-negative (hazard ratio [HR], 1.36; 95% CI, 1.34-1.38) and ER-negative/PR-positive (HR, 1.61; 95% CI, 1.55-1.67) tumors had worse BCSS than patients with the ER-positive/PR-positive subtype. In contrast, patients with ER-positive/PR-negative (HR, 1.27; 95% CI, 1.24-1.29) and ER-negative/PR-positive (HR, 1.07; 95% CI, 1.03-1.11) tumors had better BCSS than patients with the ER-negative/PR-negative subtype. The BCSS was statistically significantly worse in patients with ER-negative/PR-positive tumors than in patients with ER-positive/PR-negative tumors (HR, 1.18; 95% CI, 1.14-1.23).

**Conclusions and Relevance:**

In this cohort study, statistically significant distinctions between survival rates of patients with single hormone receptor–positive BC vs double hormone receptor–positive/double hormone receptor–negative BC were observed. Different strategies may be required for patients with single hormone receptor–positive tumors to ensure optimal treatment and maximum benefits from therapies.

## Key Points

**Question:**

Are single hormone receptor–positive tumors distinct groups of breast cancer?

**Findings:**

This cohort study analyzed 823 399 patients with breast cancer and showed that patients with single hormone receptor–positive subtypes had worse breast cancer–specific survival (BCSS) than patients with double hormone receptor–positive subtypes but better BCSS than patients with double hormone receptor–negative subtypes, and the differences were statistically significant. Patients with single estrogen receptor–positive subtypes had statistically significantly better BCSS than those with single progesterone receptor–positive subtypes.

**Meaning:**

Future studies aimed at developing optimized treatment strategies for patients with single hormone receptor–positive tumors may be warranted.

## Teaser

This cohort study investigates the clinicopathological characteristics and breast cancer–specific survival of patients with single hormone receptor–positive breast cancer.

## Introduction

Steroid hormone receptors, including estrogen receptor (ER) and progesterone receptor (PR), have been used as critical indicators for endocrine therapy and prognosis in breast cancer (BC) since the mid-1970s.^1,2^ Detection of ER and PR is recommended in patients with newly diagnosed BC in clinical practice.^3^ Compared with hormone receptor–negative BC, hormone receptor–positive BC is associated with less aggressive clinicopathological features and a better prognosis because of the benefits from endocrine therapy.^4^ The PR is encoded by an estrogen-regulated gene, and its synthesis requires estrogen and ER; therefore, ER-positive tumors are commonly PR positive, whereas ER-negative tumors are usually PR negative.^5^ However, some tumors are single hormone receptor positive (ER positive/PR negative or ER negative/PR positive) and are thought to be biologically and clinically different from double hormone receptor–positive/double hormone receptor–negative subtypes, although the cause of single hormone receptor–positive BC remains unclear.^5,6,7^ Patients with single hormone receptor–positive tumors have an intermediate prognosis between double hormone receptor–positive (best survival) and double hormone receptor–negative (worst survival) tumors, whereas there is no survival difference between patients who have ER-positive/PR-negative tumors and patients who have ER-negative/PR-positive tumors.^7^ A statistically significant difference in prognosis is only detected between single hormone receptor–positive and double hormone receptor–positive subtypes in ERBB2 (formerly HER2)–negative BC.^8^ Therefore, it is unknown whether ER-positive/PR-negative and ER-negative/PR-positive tumors are biologically and clinically distinct subtypes of BC.

Although the clinical significance of ER evaluation has been well established, the role of PR remains controversial, and whether PR assessment is necessary has been debated for years.^9,10^ The effectiveness of tamoxifen is lower in ER-positive/PR-negative tumors than in ER-positive/PR-positive tumors.^1,7,11^ Compared with patients who have ER-negative/PR-negative tumors, patients with ER-negative/PR-positive tumors can benefit from tamoxifen therapy.^12^ However, a 2011 meta-analysis^13^ of 20 randomized trials demonstrated that only ER status, but not PR status, was statistically significantly associated with tamoxifen response. Another question is whether the ER-negative/PR-positive subtype really exists or is derived from a technical artifact. Some researchers maintain that ER-negative/PR-positive tumors do not exist and believe that the ER-negative/PR-positive subtype is caused by inadequate tissue fixation or technical failure of immunohistochemical assay in ER-positive/PR-positive tumors.^14,15^ It is undeniable that the frequency of ER-negative/PR-positive tumors has decreased with the optimization of immunohistochemical techniques.^3,16^ However, studies^7,17,18^ have provided evidence that supports the biological entity of the ER-negative/PR-positive subtype, which represents a small proportion (2%-5%) of BC but has clinical characteristics different from those of the ER-positive/PR-positive subtype. These studies also confirmed the existence of the ER-negative/PR-positive phenotype using optimally fixed tissues and any level of staining as a cutoff value for positive results. In addition, PR expression can be activated through ER-independent processes, such as those involving insulin growth factor 1 and epidermal growth factor, supporting the existence of ER-negative/PR-positive tumors.^16^

However, the demographic characteristics, clinicopathological characteristics, and survival outcomes among patients with single hormone receptor–positive BC, especially ER-negative/PR-positive BC, have been poorly defined so far. In this retrospective cohort study, we investigated the clinicopathological characteristics and prognosis of single hormone receptor–positive BC based on the population from the Surveillance, Epidemiology, and End Results (SEER) database. In addition, we performed a systematic comparison between single hormone receptor–positive BC and double hormone receptor–positive/double hormone receptor–negative BC. Collectively, our findings demonstrate the distinct biological performance of ER-positive/PR-negative tumors and ER-negative/PR-positive tumors.

## Methods

### Data Source

Using SEER*Stat 8.3.5, we abstracted BC cases from the SEER data that were released in April 2018 and represent approximately 34.6% of the US population. The SEER database contains demographic, clinicopathological, treatment, and survival information. The protocol of this study was reviewed and approved by the institutional ethics committees of the First Affiliated Hospital of Chongqing Medical University, Chongqing, China. Written informed consent from patients was waived because of the public nature of the SEER database. Our analysis followed the Strengthening the Reporting of Observational Studies in Epidemiology (STROBE) reporting guidelines.^19^ The dates of analysis were September 1, 2018, to June 31, 2019.

**Figure 1.**
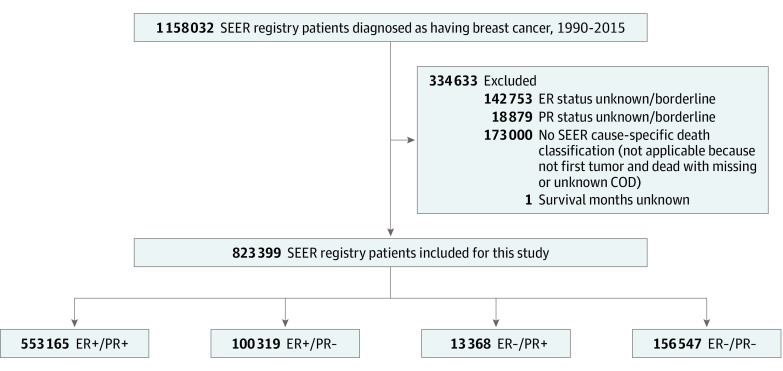
Flowchart of Patient Selection COD indicates cause of death; ER, estrogen receptor; PR, progesterone receptor; and SEER, Surveillance, Epidemiology, and End Results.

The year of diagnosis with BC was restricted to 1990 onward because information on ER and PR status was first available at that time. The latest patient population released by SEER in 2018 (the period of our analysis) comprised patients diagnosed in 2015. In total, 1 158 032 patients were originally identified. The following variables were extracted from the SEER database: year of diagnosis, age at diagnosis, sex, race, tumor size, lymph node status, distant metastasis, adjusted American Joint Committee on Cancer (AJCC) sixth edition stage, tumor grade, histology type, ERBB2 status, surgery status, chemotherapy status, radiation therapy status, survival months, and cause of death. Excluded from the analysis were patients with ER status unknown or borderline (n = 142 753), patients with PR status unknown or borderline (n = 18 879), patients without a SEER cause-specific death classification (n = 173 000), and 1 patient with survival months unknown (Figure 1). A total of 823 399 patients with BC were included in this retrospective cohort study. The BC subtypes were defined as ER positive/PR positive, ER positive/PR negative, ER negative/PR positive, and ER negative/PR negative. Patients were stratified into age groups as follows: younger than 30, 30 to 39, 40 to 49, 50 to 59, 60 to 69, 70 to 79, and 80 years or older. Race was defined according to the SEER database, and patients were stratified as white, black, or other for subgroup analysis. Patients were grouped according to tumor size as follows: less than 2 cm, 2 to 5 cm, greater than 5 cm, or unknown. According to *International Classification of Diseases for Oncology*, *Third Edition* (*ICD-O-3*) histopathology codes, histology type was classified as invasive ductal carcinoma (IDC) (*ICD-O-3* code 8500/3), invasive lobular carcinoma (ILC) (*ICD-O-3* code 8520/3), mixed IDC and ILC (*ICD-O-3* code 8522/3), or other types. The ERBB2 status was initially recorded in 2010; therefore, patients diagnosed before 2010 and those with ERBB2 status that was unknown or borderline were combined into an unknown or borderline group. Surgery status was available after 1998, and patients were grouped as yes (surgery codes 19-90), no (surgery code 00), or unknown (surgery code 99 and all cases diagnosed before 1998). The surgery codes are according to the SEER Program Coding and Staging Manual 2018.^20^ Radiation therapy codes were recorded as yes or no/unknown, including code 7 (refused) and code 8 (recommended, unknown if administered). The radiation therapy codes are according to the definition of the SEER database in the Radiation/Chemotherapy Databases (1975-2016).^21^

### Statistical Analysis

Demographic and baseline characteristics of patients with ER-positive/PR-positive, ER-positive/PR-negative, ER-negative/PR-positive, and ER-negative/PR-negative subtypes were analyzed using descriptive statistics and χ^2^ tests. Kaplan-Meier survival curves were used to assess BC-specific survival (BCSS) rates. Log-rank tests were used to examine statistical differences in survival curves. The BCSS was defined as the time from diagnosis to death from BC. Univariable Cox proportional hazards regression was performed to calculate the hazard ratios (HRs) and 95% CIs for BCSS. Multivariable Cox proportional hazards regression models were used to estimate HRs and 95% CIs for BCSS after adjusting for age at diagnosis, sex, race, tumor size, lymph node status, distant metastasis, tumor grade, and histology type. We included covariates that were defined a priori based on clinical significance and hypothesized confounders. The adjusted AJCC sixth edition stage was removed from the final models to avoid violating the principle of excluding linearly codependent variables because it was assessed by tumor size, lymph node status, and distant metastasis. To avoid bias related to missing data, ERBB2 status and treatment variables were excluded because of the large amount of missing data. The proportional hazards model assumption was confirmed for each covariate by inspecting log curves. Based on the multivariable model, HRs and 95% CIs for overall survival (OS) were also calculated. The OS was defined as the interval between diagnosis and death from any cause. All *P* values were 2-sided, and *P* < .05 was considered statistically significant. Statistical analysis was performed using IBM SPSS 22.0 software (IBM Corporation).

## Results

### Demographic and Clinicopathological Findings

**Figure 2.**
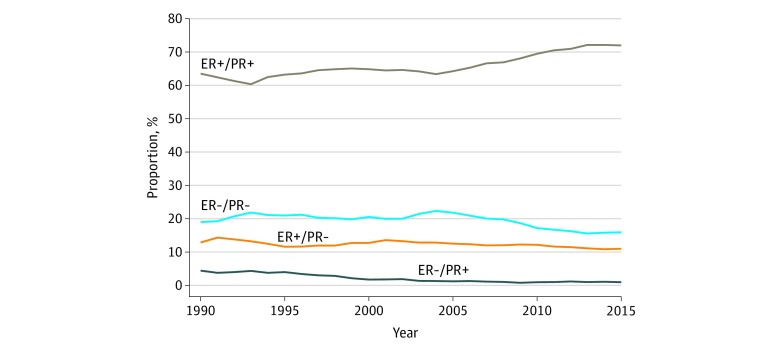
Patterns in the Proportion of Each Subtype of Breast Cancer, From the Surveillance, Epidemiology, and End Results Database, 1990-2015 ER indicates estrogen receptor; PR, progesterone receptor.

This retrospective cohort study included 823 399 patients with BC (818 002 women and 5397 men). The median (range) age at diagnosis was 60 (8-108) years, and the median (range) follow-up duration was 71 (0-311) months. The demographic and clinicopathological characteristics of patients are listed in Table 1. Among the included patients, the percentages of ER-positive/PR-positive, ER-positive/PR-negative, ER-negative/PR-positive, and ER-negative/PR-negative cases were 67.2%, 12.2%, 1.6%, and 19.0%, respectively. Between 1990 and 2015, the proportion of patients with ER-positive/PR-negative disease decreased from 12.9% to 11.0% (decreased by 1.9%), ER-negative/PR-positive disease decreased from 4.5% to 1.0% (decreased by 3.5%), and ER-negative/PR-negative disease decreased from 19.0% to 15.9% (decreased by 3.1%), whereas the rate of ER-positive/PR-positive disease increased from 63.6% to 72.1% (decreased by 8.5%) (Figure 2). In the most recent 5 years between 2011 and 2015, the proportions of ER-positive/PR-positive, ER-positive/PR-negative, ER-negative/PR-positive, and ER-negative/PR-negative tumors were stabilized at approximately 72%, 11%, 1%, and 16%, respectively.

**Table 1.** Characteristics of Patients With Breast Cancer Stratified by Hormone Receptor Status, From the Surveillance, Epidemiology, and End Results Database, 1990-2015^a^

Characteristic	No./Total No. (%)				
	All Patients (N = 823 399)	ER+/PR+ (n = 553 165)	ER+/PR− (n = 100 319)	ER−/PR+ (n = 13 368)	ER−/PR− (n = 156 547)
Age at diagnosis, median (range), y	60 (8-108)	61 (17-107)	62 (8-108)	53 (19-104)	56 (10-105)
Age group at diagnosis, y					
<30	4800 (0.6)	2330 (0.4)	524 (0.5)	149 (1.1)	1797 (1.1)
30-39	43 781 (5.3)	24 023 (4.3)	4328 (4.3)	1365 (10.2)	14 065 (9.0)
40-49	152 492 (18.5)	102 812 (18.6)	12 264 (12.2)	3676 (27.5)	33 740 (21.6)
50-59	203 933 (24.8)	131 427 (23.8)	25 850 (25.8)	3415 (25.5)	43 241 (27.6)
60-69	196 670 (23.9)	135 831 (24.6)	26 027 (25.9)	2398 (17.9)	32 414 (20.7)
70-79	143 324 (17.4)	101 802 (18.4)	19 507 (19.4)	1573 (11.8)	20 442 (13.1)
≥80	78 399 (9.5)	54 940 (9.9)	11 819 (11.8)	792 (5.9)	10 848 (6.9)
Sex, No. (%)					
Male	5397 (0.7)	4570 (0.8)	542 (0.5)	50 (0.4)	235 (0.2)
Female	818 002 (99.3)	548 595 (99.2)	99 777 (99.5)	13 318 (99.6)	156 312 (99.8)
Race					
White	668 102/819 361 (81.5)	459 856/550 273‬ (83.6)	81 126/99 864‬ (81.2)	10 200/13 308 (76.6)	116 920/155 916 (75.0)
Black	83 371/819 361 (10.2)	44 235/550 273‬ (8.0)	10 953/99 864‬ (11.0)	1902/13 308 (14.3)	26 281/155 916 (16.9)
Other	67 888/819 361 (8.3)	46 182/550 273‬ (8.4)	7785/99 864‬ (7.8)	1206/13 308 (9.1)	12 715/155 916 (8.2)
Unknown, No.	4038	2892	455	60	631
Tumor size, cm					
<2	423 898/771 918 (54.9)	313 287/524 472‬ (59.7)	48 337/92 945‬ (52.0)	5480/12 088 (45.3)	56 794/142 413‬ (39.9)
2-5	292 876/771 918 (37.9)	180 867/524 472‬ (34.5)	36 696/92 945‬ (39.5)	5483/12 088 (45.4)	69 830/142 413‬ (49.0)
>5	55 144/771 918 (7.1)	30 318/524 472‬ (5.8)	7912/92 945‬ (8.5)	1125/12 088 (9.3)	15 789/142 413‬ (11.1)
Unknown, No.	51 481	28 693	7374	1280	14 134
Lymph node status					
Positive	270 137/780 835‬ (34.6)	171 865/526 696 (32.6)	34 187/94 353‬ (36.2)	4909/12 428 (39.5)	59 176/147 358‬ (40.2)
Negative	510 698/780 835‬ (65.4)	354 831/526 696 (67.4)	60 166/94 353‬ (63.8)	7519/12 428 (60.5)	88 182/147 358‬ (59.8)
Unknown, No.	42 564	26 469	5966	940	9189
Distant metastasis					
M0	777 844/812 878 (95.7)	526 944/546 840 (96.4)	93 074/98 872 (94.1)	12 464/13 183 (94.5)	145 362/153 983 (94.4)
M1	35 034/812 878 (4.3)	19 896/546 840 (3.6)	5798/98 872 (5.9)	719/13 183 (5.5)	8621/153 983 (5.6)
Unknown, No.	10 521	6325	1447	185	2564
Adjusted AJCC sixth edition stage					
0	589/788 671‬ (0.1)	118/531 107 (0.0)	110/95 678 (0.1)	18/12 620 (0.1)	343/149 266 (0.2)
I	370 170/788 671‬ (46.9)	272 254/531 107 (51.3)	42 494/95 678 (44.4)	4807/12 620 (38.1)	50 615/149 266 (33.9)
II	278 214/788 671‬ (35.3)	178 453/531 107 (33.6)	33 113/95 678 (34.6)	4869/12 620 (38.6)	61 779/149 266 (41.4)
III	104 664/788 671‬ (13.3)	60 386/531 107 (11.4)	14 163/95 678 (14.8)	2207/12 620 (17.5)	27 908/149 266 (18.7)
IV	35 034/788 671‬ (4.4)	19 896/531 107 (3.7)	5798/95 678 (6.1)	719/12 620 (5.7)	8621/149 266 (5.8)
Unknown, No.	34 728	22 058	4641	748	7281
Tumor grade					
I	156 063/756 167‬ (20.6)	135 287/509 817‬ (26.5)	16 269/90 602 (18.0)	864/11 866 (7.3)	3643/143 882 (2.5)
II	322 437/756 167‬ (42.6)	253 316/509 817‬ (49.7)	38 301/90 602 (42.3)	3212/11 866 (27.1)	27 608/143 882 (19.2)
III	267 554/756 167‬ (35.4)	117 022/509 817‬ (23.0)	34 853/90 602 (38.5)	7454/11 866 (62.8)	108 225/143 882 (75.2)
IV	10 113/756 167‬ (1.3)	4192/509 817‬ (0.8)	1179/90 602 (1.3)	336/11 866 (2.8)	4406/143 882 (3.1)
Unknown, No.	67 232	43 348	9717	1502	12 665
Histology type, No. (%)^b^					
IDC, code 8500/3	603 203 (73.3)	394 003 (71.2)	71 228 (71.0)	10 409 (77.9)	127 563 (81.5)
ILC, code 8520/3	68 336 (8.3)	54 349 (9.8)	10 913 (10.9)	569 (4.3)	2505 (1.6)
Mixed IDC and ILC, code 8522/3	54 061 (6.6)	43 948 (7.9)	6577 (6.6)	516 (3.9)	3020 (1.9)
Other types	97 799 (11.9)	60 865 (11.0)	11 601 (11.6)	1874 (14.0)	23 459 (15.0)
ERBB2 status					
Positive	43 845/277 691 (15.8)	21 669/198 143 (10.9)	7943/31 409 (25.3)	911/2934 (31.0)	13 322/45 205 (29.5)
Negative	233 846/277 691 (84.2)	176 474/198 143 (89.1)	23 466/31 409 (74.7)	2023/2934 (69.0)	31 883/45 205 (70.5)
Unknown or borderline, No.	545 708	355 022	68 910	10 434	111 342
Surgery status					
Yes	671 516/716 907 (93.7)	459 120/486 214 (94.4)	79 987/86 857 (92.1)	8581/9315 (92.1)	123 828/134 521 (92.1)
No	45 391/716 907 (6.3)	27 094/486 214 (5.6)	6870/86 857 (7.9)	734/9315 (7.9)	10 693/134 521 (7.9)
Unknown, No.	106 492	66 951	13 462	4053	22 026
Chemotherapy status					
Yes	339 415 (41.2)	185 752 (33.6)	42 416 (42.3)	7754 (58.0)	103 493 (66.1)
No or unknown	483 984 (58.8)	367 413 (66.4)	57 903 (57.7)	5614 (42.0)	53 054 (33.9)
Radiation therapy status					
Yes	411 436 (50.0)	283 848 (51.3)	48 389 (48.2)	6134 (45.9)	73 065 (46.7)
No or unknown	411 963 (50.0)	269 317 (48.7)	51 930 (51.8)	7234 (54.1)	83 482 (53.3)
Vital status					
Alive	591 558 (71.8)	414 154 (74.9)	66 398 (66.2)	8472 (63.4)	102 534 (65.5)
Died of breast cancer	115 524 (14.0)	58 485 (10.6)	17 371 (17.3)	3104 (23.2)	36 564 (23.4)
Died of other cause	116 317 (14.1)	80 526 (14.6)	16 550 (16.5)	1792 (13.4)	17 449 (11.1)

Abbreviations: AJCC, American Joint Committee on Cancer; ER, estrogen receptor; IDC, invasive ductal carcinoma; ILC, invasive lobular carcinoma; PR, progesterone receptor.

^a^ *P* < .001 for all comparisons, calculated using the Pearson χ^2^ test.

^b^ By *International Classification of Diseases for Oncology*, *Third Edition* histopathology code.

Compared with other groups, ER-positive/PR-negative tumors were more frequent in individuals 60 years or older (57.1%), whereas ER-negative/PR-positive tumors were more frequent in individuals aged 30 to 49 years (37.7%). Sex and race differed statistically significantly among the 4 subtypes. The ER-positive/PR-positive group included the highest proportion of patients with less aggressive tumor characteristics, including tumor size smaller than 2 cm, negative lymph node status, adjusted AJCC sixth edition stage I, and tumor grade I to II, and the proportions of these patients gradually decreased from ER-positive/PR-positive to ER-positive/PR-negative to ER-negative/PR-positive to ER-negative/PR-negative tumors (Table 1). In contrast, the proportions of patients with more aggressive tumor characteristics, including tumor size of at least 2 cm, positive lymph node status, adjusted AJCC sixth edition stage II to III, and tumor grade III to IV, gradually increased from ER-positive/PR-positive to ER-positive/PR-negative to ER-negative/PR-positive to ER-negative/PR-negative tumors (Table 1). Invasive ductal carcinoma was more common in ER-negative/PR-positive (77.9%) and ER-negative/PR-negative (81.5%) tumors, whereas ILC and mixed IDC/ILC were more common in ER-positive/PR-positive (17.7%) and ER-positive/PR-negative (17.5%) tumors. The ER-negative/PR-positive group (31.0%) had the highest proportion of ERBB2-positive tumors; however, there was no difference between ER-negative/PR-positive and ER-negative/PR-negative subtypes with respect to ERBB2 status.

For surgery status, there was no difference between patients with ER-positive/PR-negative, ER-negative/PR-positive, and ER-negative/PR-negative tumors. However, the results for the subgroup analyses of ERBB2 status and surgery status should be interpreted with caution because of the large proportions of patients with ERBB2 status unknown or borderline (66.3% [n = 545 708]) and surgery status unknown (12.8% [n = 105 648]). Chemotherapy was administered more often to ER-negative/PR-negative tumors (66.1%), followed by ER-negative/PR-positive (58.0%), ER-positive/PR-negative (42.3%), and ER-positive/PR-positive (33.6%) tumors. For radiation therapy, no difference was observed between the ER-negative/PR-positive and ER-negative/PR-negative subtypes. Patients with ER-negative/PR-positive and ER-negative/PR-negative subtypes had higher rates of death from BC than other subtypes, whereas patients with ER-positive/PR-negative subtype were most likely to die of other causes.

### Survival Analysis

**Figure 3.**
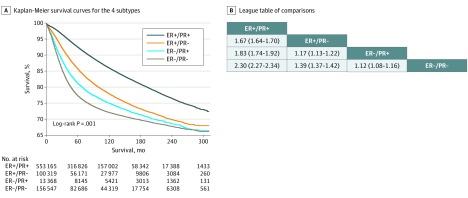
Breast Cancer–Specific Survival of Patients Stratified by Estrogen Receptor (ER) Status and Progesterone Receptor (PR) Status A, Kaplan-Meier survival curves show breast cancer–specific survival rates. B, Data are presented as hazard ratios (95% CIs). A hazard ratio exceeding 1 favors the column-defining subtype.

The BCSS differed statistically significantly among the 4 subtypes (Figure 3A). The estimated mean BCSS was 261.3 (95% CI, 260.9-261.7) months in patients with the ER-positive/PR-positive subtype, 241.2 (95% CI, 240.2-242.2) months in those with the ER-positive/PR-negative subtype, 233.6 (95% CI, 231.3-235.9) months in those with the ER-negative/PR-positive subtype, and 227.6 (95% CI, 226.8-228.5) months in those with the ER-negative/PR-negative subtype. Patients with ER-positive/PR-positive tumors had the highest 10-year and 20-year BCSS, followed by ER-positive/PR-negative, ER-negative/PR-positive, and ER-negative/PR-negative tumors (eTable 1 in the Supplement). In the unadjusted analysis, the ER-positive/PR-negative (HR, 1.67; 95% CI, 1.64-1.70) and ER-negative/PR-positive (HR, 1.83; 95% CI, 1.74-1.92) subtypes had a higher risk of BC-specific death than the ER-positive/PR-positive subtype (Figure 3B). However, both the ER-positive/PR-negative (HR, 1.39; 95% CI, 1.37-1.42) and ER-negative/PR-positive (HR, 1.12; 95% CI, 1.08-1.16) subtypes were statistically significantly associated with better BCSS than the ER-negative/PR-negative subtype. Patients with ER-negative/PR-positive tumors had poorer BCSS than patients with ER-positive/PR-negative tumors (HR, 1.17; 95% CI, 1.13-1.22).

Subgroup analyses were performed based on sex, race, adjusted AJCC sixth edition stage, tumor grade, histology type, and ERBB2 status (eFigures 1-6 in the Supplement). The BCSS was best for the ER-positive/PR-positive subtype, followed by ER-positive/PR-negative, ER-negative/PR-positive, and ER-negative/PR-negative subtypes in female, white, adjusted AJCC sixth edition stage 0 to II, and IDC subgroups (eTable 2 and eFigures 1-4 in the Supplement). However, no statistically significant BCSS difference was observed between ER-positive/PR-negative and ER-negative/PR-positive subtypes in male, black, tumor grade II, adjusted AJCC sixth edition stage IV, ILC, or mixed IDC/ILC subgroups (eTable 2 in the Supplement). There was no statistically significant difference in BCSS between patients with ER-negative/PR-positive and ER-negative/PR-positive tumors in the subgroups of male, other race, adjusted AJCC sixth edition stages III and IV, tumor grades III and IV, other histology types, or any ERBB2 status (eTable 2 in the Supplement). Although there was no statistically significant BCSS difference between ER-negative/PR-positive and ER-negative/PR-negative subtypes in patients with adjusted AJCC sixth edition stages III and IV disease, the estimated median BCSS of the ER-negative/PR-positive subtype (60.0 months; 95% CI, 53.4-66.6 months) was longer than that of the ER-negative/PR-negative subtype (47.0 months; 95% CI, 45.5-48.5 months). We subsequently investigated the association between ERBB2 status and BCSS in each hormone receptor subgroup (eFigure 7 in the Supplement). Compared with positive ERBB2 status, negative ERBB2 status was statistically significantly associated with better BCSS in the ER-positive/PR-positive subtype and with worse BCSS in the ER-negative/PR-positive and ER-negative/PR-negative subtypes. The BCSS did not differ statistically significantly between ERBB2-negative and ERBB2-positive tumors in ER-positive/PR-negative patients. There was a statistically significant BCSS difference among the 4 subtypes in patients diagnosed between 1990 and 2004, whereas no BCSS difference was detected between the ER-negative/PR-positive and ER-negative/PR-negative subtypes for patients diagnosed between 2005 and 2015 (eTable 2 and eFigure 8 and in the Supplement).

Among patients who underwent surgery and radiation therapy, the ER-positive/PR-positive patients had the best BCSS, followed by the ER-positive/PR-negative, ER-negative/PR-positive, and ER-negative/PR-negative patients (eFigures 9 and 10 in the Supplement). However, in patients who did not undergo surgery, there was no difference in BCSS between the ER-negative/PR-positive and ER-negative/PR-negative subtypes. Among patients who received chemotherapy, the 10-year BCSS was 81.2%, 72.3%, 71.6%, and 70.6% for the ER-positive/PR-positive, ER-positive/PR-negative, ER-negative/PR-positive, and ER-negative/PR-negative subtypes, respectively (eTable 1 in the Supplement). However, the BCSS of the ER-negative/PR-positive and ER-negative/PR-negative subtypes gradually exceeded that of the ER-positive/PR-negative subtype at approximately 150 months after diagnosis (eFigure 11 in the Supplement).

In multivariable analysis, compared with the ER-positive/PR-positive subtype, the ER-positive/PR-negative, ER-negative/PR-positive, and ER-negative/PR-negative subtypes were significantly associated with a 36% (HR, 1.36; 95% CI, 1.34-1.38), 61% (HR, 1.61; 95% CI, 1.55-1.67), and 72% (HR, 1.72; 95% CI, 1.70-1.75) increased risk of BC-specific death, respectively (Table 2). When ER-positive/PR-negative subtype was set as reference, patients with ER-negative/PR-positive (HR, 1.18; 95% CI, 1.14-1.23) and ER-negative/PR-negative (HR, 1.27; 95% CI, 1.24-1.29) subtypes had an 18% and 27% higher risk of BC-specific death, respectively (eTable 3 in the Supplement). Patients with the ER-negative/PR-negative subtype had a 7% higher risk of BC-specific death (HR, 1.07; 95% CI, 1.03-1.11) than those with the ER-negative/PR-positive subtype.

**Table 2.** Multivariable Cox Proportional Hazards Regression Analysis for BCSS and OS, From the Surveillance, Epidemiology, and End Results Database, 1990-2015^a^

Variable	BCSS		OS	
	HR (95% CI)	*P* Value	HR (95% CI)	*P* Value
Age group at diagnosis, y				
<30	1 [Reference]	NA	1 [Reference]	NA
30-39	0.94 (0.88-1.00)	.04	0.88 (0.83-0.93)	<.001
40-49	0.82 (0.77-0.87)	<.001	0.76 (0.72-0.81)	<.001
50-59	0.89 (0.83-0.94)	<.001	0.97 (0.92-1.03)	.34
60-69	1.03 (0.97-1.09)	.42	1.56 (1.47-1.65)	<.001
70-79	1.38 (1.30-1.47)	<.001	3.13 (2.96-3.32)	<.001
≥80	2.27 (2.13-2.41)	<.001	6.84 (6.45-7.26)	<.001
Sex				
Male	1 [Reference]	NA	1 [Reference]	NA
Female	0.80 (0.75-0.85)	<.001	0.72 (0.69-0.75)	<.001
Race				
White	1 [Reference]	NA	1 [Reference]	NA
Black	1.37 (1.35-1.39)	<.001	1.36 (1.34-1.38)	<.001
Other	0.87 (0.85-0.89)	<.001	0.82 (0.80-0.83)	<.001
Unknown	0.24 (0.20-0.30)	<.001	0.25 (0.21-0.29)	<.001
Tumor size, cm				
<2	1 [Reference]	NA	1 [Reference]	NA
2-5	1.99 (1.96-2.02)	<.001	1.46 (1.45-1.48)	<.001
>5	3.01 (2.95-3.08)	<.001	2.15 (2.11-2.18)	<.001
Unknown	2.52 (2.47-2.58)	<.001	1.59 (1.56-1.61)	<.001
Lymph node status				
Negative	1 [Reference]	NA	1 [Reference]	NA
Positive	2.39 (2.36-2.42)	<.001	1.57 (1.56-1.59)	<.001
Unknown	2.03 (1.98-2.08)	<.001	1.53 (1.50-1.55)	<.001
Distant metastasis				
M0	1 [Reference]	NA	1 [Reference]	NA
M1	7.59 (7.47-7.71)	<.001	5.71 (5.62-5.79)	<.001
Unknown	1.79 (1.72-1.87)	<.001	1.51 (1.46-1.56)	<.001
Tumor grade				
I	1 [Reference]	NA	1 [Reference]	NA
II	1.71 (1.67-1.76)	<.001	1.16 (1.14-1.18)	<.001
III	2.44 (2.37-2.50)	<.001	1.43 (1.41-1.45)	<.001
IV	2.50 (2.39-2.62)	<.001	1.42 (1.37-1.46)	<.001
Unknown	2.08 (2.02-2.14)	<.001	1.32 (1.29-1.34)	<.001
Histology type^b^				
IDC, code 8500/3	1 [Reference]	NA	1 [Reference]	NA
ILC, code 8520/3	1.02 (1.00-1.04)	.11	0.94 (0.93-0.95)	<.001
Mixed IDC and ILC, code 8522/3	1.00 (0.97-1.02)	.88	0.93 (0.91-0.94)	<.001
Other types	0.99 (0.97-1.01)	.26	1.00 (0.99-1.02)	.68
Hormone receptor				
ER+/PR+	1 [Reference]	NA	1 [Reference]	NA
ER+/PR−	1.36 (1.34-1.38)	<.001	1.19 (1.17-1.20)	<.001
ER−/PR+	1.61 (1.55-1.67)	<.001	1.27 (1.23-1.30)	<.001
ER−/PR−	1.72 (1.70-1.75)	<.001	1.36 (1.35-1.38)	<.001

Abbreviations: BCSS, breast cancer–specific survival; ER, estrogen receptor; HR, hazard ratio; IDC, invasive ductal carcinoma; ILC, invasive lobular carcinoma; NA, not applicable; OS, overall survival; PR, progesterone receptor.

^a^ Adjusted for age at diagnosis, sex, race, tumor size, lymph node status, distant metastasis, tumor grade, and histology type.

^b^ By *International Classification of Diseases for Oncology*, *Third Edition* histopathology code.

Regarding OS, patients with ER-positive/PR-negative, ER-negative/PR-positive, and ER-negative/PR-negative tumors had statistically significantly poorer OS compared with patients with the ER-positive/PR-positive subtype (Table 2). Patients with ER-negative/PR-positive tumors had a 7% higher risk of all-cause death (HR, 1.07; 95% CI, 1.04-1.10) and those with ER-negative/PR-negative tumors had a 15% (HR, 1.15; 95% CI, 1.13-1.17) higher risk of all-cause death than those with ER-positive/PR-negative tumors, whereas patients with ER-negative/PR-negative tumors had an 8% (HR, 1.08; 95% CI, 1.06-1.11) higher risk of all-cause death than those with ER-negative/PR-positive tumors (eTable 3 in the Supplement).

## Discussion

The steroid hormone receptors ER and PR are 2 critical molecules for assessing the heterogeneity of BC and the benefit of therapy.^22^ However, to date, the understanding of the clinical significance of hormone receptor status, especially in single hormone receptor–positive tumors, has been poorly investigated because of limited sample sizes. The present study analyzed 823 399 patients with BC, including 100 319 ER-positive/PR-negative cases and 13 368 ER-negative/PR-positive cases. To our knowledge, this represents the largest number of patients with single hormone receptor–positive BC analyzed to date. We found that the proportions of the 4 subtypes statistically significantly varied over the past 26 years. The proportion of patients with ER-positive/PR-positive tumors increased by 8.5%, whereas the proportions of patients with ER-positive/PR-negative, ER-positive/PR-negative, and ER-negative/PR-positive tumors decreased by 1.9%, 3.5%, and 3.1%, respectively. We propose 2 possible reasons for these changes. The incidence of the 4 subtypes may have changed over time. This is supported by the observation that the number of hormone receptor–positive BC cases increased from 1992 to 1998 among patients aged 40 to 69 years,^23^ a pattern that may be caused by hormonal factors, such as early age at menarche, higher body mass index, and increased use of hormone therapy.^23,24^ Alternatively, tumors that might be detected as ER negative and/or PR negative may have subsequently been classified as ER positive and/or PR positive with the development of immunohistochemical techniques. However, in the 5 years between 2011 and 2015, the proportions of ER-positive/PR-positive, ER-positive/PR-negative, ER-negative/PR-positive, and ER-negative/PR-negative tumors stabilized at approximately 72%, 11%, 1%, and 16%, respectively, which implies the existence of the ER-negative/PR-positive subtype regardless of the incidence and use of immunohistochemical techniques.

Estrogen receptor–positive/PR-negative tumors are more common in older and postmenopausal women.^7,8,25,26^ In this study, ER-positive/PR-negative tumors were most frequent in individuals 60 years or older, whereas ER-negative/PR-positive tumors were most frequent in individuals aged 30 to 49 years. Moreover, we found statistically significant differences in sex and race among the 4 subtypes, a finding that has not been previously reported, to our knowledge. In 2007, Rakha et al^7^ reported that patients with single hormone receptor–positive BC have more advanced clinicopathological features than patients with ER-positive/PR-positive subtypes and that patients with single hormone receptor–positive BC have favorable features compared with the ER-negative/PR-negative subtype; however, no difference in outcomes was detected between patients with ER-positive/PR-negative tumors and those with ER-negative/PR-positive tumors regarding lymph node status and BC survival. In the present study, we found a similar phenomenon in that patients with the single hormone receptor–positive subtype had more aggressive clinicopathological features, including larger tumor size, positive lymph node status, distant metastasis, adjusted AJCC sixth edition advanced stage, higher tumor grade, and BC-specific death, than those with the ER-positive/PR-positive subtype, but those with the single hormone receptor–positive subtype had less aggressive clinicopathological features than those with ER-negative/PR-negative subtype. Moreover, ER loss (ie, ER negative/PR positive) was associated with more aggressive tumor behavior than PR loss (ie, ER positive/PR negative). The ERBB2 status among the 4 subtypes described herein differed from that in previous studies.^7,8,27^ The limited number of cases in those studies may explain this difference. However, ERBB2 status in patients with single hormone receptor–positive BC in our analysis was consistent with that reported in a study^27^ with a large sample size, which showed that ERBB2 status differed statistically significantly among the 4 subtypes. These results suggest that ER-positive/PR-negative and ER-negative/PR-positive tumors represent 2 biological subtypes distinct from ER-positive/PR-positive and ER-negative/PR-negative BC, although ER-negative/PR-positive tumors are rare.

Patients with the ER-positive/PR-positive subtype had a better prognosis than patients with the ER-negative/PR-negative subtype; however, the survival outcomes of single hormone receptor–positive BC are unknown.^5,7,28,29^ Although the findings of a 2001 study^28^ based on the SEER database suggested that BCSS ranks from good to worse for ER-positive/PR-positive, ER-positive/PR-negative, ER-negative/PR-positive, and ER-negative/PR-negative subtypes, that study only included 19 541 non-Hispanic white women with node-negative BC and lacked long-term follow-up information, which weakens the robustness of the results. However, a 2007 study by Rakha et al^7^ found no statistically significant survival difference between ER-positive/PR-negative and ER-negative/PR-positive subtypes or between ER-negative/PR-positive and ER-negative/PR-negative subtypes. In 2015, Bae et al^8^ reported that ER-positive/PR-negative and ER-negative/PR-positive tumors are associated with poorer disease-free survival and OS than ER-positive/PR-positive tumors, but they found that ER-positive/PR-negative and ER-negative/PR-positive tumors are associated with poor survival similar to that of the ER-negative/PR-negative subtype in ERBB2-negative BC; there was no difference in survival among the 4 subtypes in patients with ERBB2-positive disease.

In this study, we found a statistically significant BCSS difference not only between ER-positive/PR-negative and ER-negative/PR-positive tumors but also between single hormone receptor–positive and ER-negative/PR-negative subtypes in the overall cohort. Our results are consistent with and further strengthen the evidence provided by Anderson et al.^28^ However, only a modest or no BCSS difference was detected in the present study in the comparison among the ER-positive/PR-negative, ER-negative/PR-positive, and ER-negative/PR-negative subtypes in several subgroups with a small number of patients, including men, black individuals, and those with grade IV BC. After stratifying patients into ERBB2-positive and ERBB2-negative groups, we found a statistically significant BCSS difference among the 4 subtypes, except when comparing the ER-negative/PR-positive subtype with the ER-negative/PR-negative subtype in ERBB2-negative and ERBB2-positive patients. Multivariable analysis demonstrated that the 4 subtypes (ER positive/PR positive, ER positive/PR negative, ER negative/PR positive, and ER negative/PR negative) are independent factors of BCSS and OS. Consistent with the findings by Rakha et al,^7^ our findings suggest that single hormone receptor–positive BC has a prognosis midway between that of the ER-positive/PR-positive and ER-negative/PR-negative subtypes; however, we also observed that ER loss appeared to be statistically significantly associated with worse BCSS and OS than PR loss. Specifically, ER loss increased the risk of BC-specific death by 18% and the risk of all-cause death by 7% compared with PR loss. Accordingly, the associated patterns of the 4 subtypes are distinct from each other.

Our findings suggest that adjuvant endocrine therapy should be limited to hormone receptor-positive BC based on evidence demonstrating that additional hormone therapy is not beneficial in patients with the ER-negative/PR-negative subtype.^30^ However, the value of single hormone receptor–positivity for assessing the benefit from endocrine therapy remains unknown. Tamoxifen therapy is believed to be more beneficial in patients with ER-positive/PR-positive tumors than in patients with ER-positive/PR-negative tumors.^5,11^ Dowsett et al^31^ identified a strong trend in the benefit from tamoxifen therapy for patients with ER-negative/PR-positive tumors. In ER-negative/PR-positive ERBB2-negative BC, a recent retrospective study^32^ found that patients who receive adjuvant endocrine therapy have statistically significantly longer relapse-free survival and OS than patients who do not receive endocrine therapy. These results suggest that the PR contributes to the response to endocrine therapy, although the ER might have a more important role in endocrine therapy than the PR. In the study by Kunc et al,^16^ patients with ER-negative/PR-positive tumors derived less benefit from endocrine therapy than those with ER-positive/PR-positive tumors; however, the opposite was observed for chemotherapy. The final analysis of the Chemotherapy as Adjuvant for Locally Recurrent Breast Cancer (CALOR) trial^33^ showed that patients with ER-negative isolated locoregional BC recurrence had a statistically significant benefit from chemotherapy, whereas no benefit was detected for patients with ER-positive isolated locoregional BC recurrence. In the present study, we observed that adjuvant chemotherapy might alter the BCSS pattern of the 4 subtypes, with differences in the benefit from chemotherapy: after approximately 12.5 years, the BCSS of patients with ER-negative/PR-negative and ER-negative/PR-positive tumors gradually exceeded that of patients with the ER-positive/PR-negative subtype. Our results are supported in part by evidence from a meta-analysis^34^ finding that the absolute benefit from chemotherapy is greater for ER-negative BC than for ER-positive BC. Therefore, the combination of endocrine therapy and chemotherapy might be a rational treatment for ER-negative/PR-positive disease.

### Limitations

This study has limitations. First, the definition of ER-negative BC or PR-negative BC is uncertain. Since 2010, the guidelines for immunohistochemical analysis have defined negativity as less than 1% of tumor cells with positive ER or PR staining.^3^ However, during our study period, the cutoff for defining hormone receptor negativity was not uniform, and it remains unclear whether the cutoff included a range of 1% to 10% or more in some SEER registries.^35^ Therefore, it would be more appropriate to consider ER-negative and PR-negative status as less than 10% of positively stained cells when interpreting the results of this study. Second, 334 633 patients were excluded because of the lack of crucial information; therefore, potential selection bias could not be avoided. Third, we were unable to investigate the benefit from endocrine therapy for single hormone receptor–positive subtypes because information on endocrine therapy was not available. Fourth, we could not further comment on the consequences of chemotherapy and radiation therapy because detailed data were not recorded in the SEER database. Fifth, although ERBB2 status was available after 2010, the follow-up period for most patients was less than 6 years, which might have disguised the survival difference between ER-negative/PR-positive and ER-negative/PR-negative tumors when stratifying patients according to ERBB2 status. Hence, future studies are needed to investigate the long-term follow-up results in these patients.

## Conclusions

This study represents the largest analysis to date, to our knowledge, focusing on single hormone receptor–positive BC. We provide novel insights into the epidemiology of single hormone receptor–positive subtypes. The results support the existence of the ER-negative/PR-positive subtype and indicate that ER-positive/PR-negative and ER-negative/PR-positive tumors are distinct subtypes of BC. The assessment of PR status provides valuable information for prognosis and for evaluating the benefit of endocrine therapy. Further studies and clinical trials are needed to optimize the treatment regimens for patients with single hormone receptor–positive BC.

## Notes

Supplement. **eTable 1.** Median or Mean Survival (Months) and 10- and 20-Year Breast Cancer-Specific Survival in Each Subtype, SEER Data Source, 1990-2015 **eTable 2.** Hazard Ratios and Log-Rank *P* Values of Breast Cancer-Specific Survival in Subgroup Analyses **eTable 3.** Multivariable Cox Regression Analysis of Breast Cancer-Specific Survival (BCSS) and Overall Survival (OS), SEER Data Source, 1990-2015 **eFigure 1.** Breast Cancer-Specific Survival Stratified by Sex **eFigure 2.** Breast Cancer-Specific Survival Stratified by Race **eFigure 3.** Breast Cancer-Specific Survival Stratified by AJCC Stage **eFigure 4.** Breast Cancer-Specific Survival Stratified by Pathological Grade **eFigure 5.** Breast Cancer-Specific Survival Stratified by Histology **eFigure 6.** Breast Cancer-Specific Survival Stratified by ERBB2 Status **eFigure 7.** Breast Cancer-Specific Survival of ERBB2-Positive and ERBB2-Negative Subgroups in Each Hormone Receptor Subgroup **eFigure 8.** Breast Cancer-Specific Survival Stratified by Year of Diagnosis **eFigure 9.** Breast Cancer-Specific Survival Stratified by Patients Who Received Surgery or Not **eFigure 10.** Breast Cancer-Specific Survival of Patients Who Received Radiotherapy **eFigure 11.** Breast Cancer-Specific Survival of Patients Who Received Chemotherapy
