# Supplementary material for: Clinicopathological Characteristics and Breast Cancer–Specific Survival of Patients With Single Hormone Receptor–Positive Breast Cancer
Source: JAMA Netw Open. 2020 Jan 3;3(1):e1918160. doi: 10.1001/jamanetworkopen.2019.18160 (PMC6991239; doi:10.1001/jamanetworkopen.2019.18160)
Supplement: Supplement. — eTable 1. Median or Mean Survival (Months) and 10- and 20-Year Breast Cancer-Specific Survival in Each Subtype, SEER Data Source, 1990-2015 eTable 2. Hazard Ratios and Log-Rank P Values of Breast Cancer-Specific Survival in Subgroup Analyses eTable 3. Multivariable Cox Regression Analysis of Breast Cancer-Specific Survival (BCSS) and Overall Survival (OS), SEER Data Source, 1990-2015 eFigure 1. Breast Cancer-Specific Survival Stratified by Sex eFigure 2. Breast Cancer-Specific Survival Stratified by Race eFigure 3. Breast Cancer-Specific Survival Stratified by AJCC Stage eFigure 4. Breast Cancer-Specific Survival Stratified by Pathological Grade eFigure 5. Breast Cancer-Specific Survival Stratified by Histology eFigure 6. Breast Cancer-Specific Survival Stratified by ERBB2 Status eFigure 7. Breast Cancer-Specific Survival of ERBB2-Positive and ERBB2-Negative Subgroups in Each Hormone Receptor Subgroup eFigure 8. Breast Cancer-Specific Survival Stratified by Year of Diagnosis eFigure 9. Breast Cancer-Specific Survival Stratified by Patients Who Received Surgery or Not eFigure 10. Breast Cancer-Specific Survival of Patients Who Received Radiotherapy eFigure 11. Breast Cancer-Specific Survival of Patients Who Received Chemotherapy [file jamanetwopen-3-e1918160-s001.pdf]

Supplementary Online Content

Li Y, Yang D, Yin X, et al. Clinicopathological characteristics and breast cancer–specific survival of patients with single hormone receptor–positive breast cancer. *JAMA Netw Open*. 2020;3(1):e1918160. doi:10.1001/jamanetworkopen.2019.18160

eTable 1. Median or Mean Survival (Months) and 10- and 20-Year Breast Cancer-Specific Survival in Each Subtype, SEER Data Source, 1990-2015
eTable 2. Hazard Ratios and Log-Rank *P* Values of Breast Cancer-Specific Survival in Subgroup Analyses
eTable 3. Multivariable Cox Regression Analysis of Breast Cancer-Specific Survival (BCSS) and Overall Survival (OS), SEER Data Source, 1990-2015
eFigure 1. Breast Cancer-Specific Survival Stratified by Sex
eFigure 2. Breast Cancer-Specific Survival Stratified by Race
eFigure 3. Breast Cancer-Specific Survival Stratified by AJCC Stage
eFigure 4. Breast Cancer-Specific Survival Stratified by Pathological Grade
eFigure 5. Breast Cancer-Specific Survival Stratified by Histology
eFigure 6. Breast Cancer-Specific Survival Stratified by ERBB2 Status
eFigure 7. Breast Cancer-Specific Survival of ERBB2-Positive and ERBB2-Negative Subgroups in Each Hormone Receptor Subgroup
eFigure 8. Breast Cancer-Specific Survival Stratified by Year of Diagnosis
eFigure 9. Breast Cancer-Specific Survival Stratified by Patients Who Received Surgery or Not
eFigure 10. Breast Cancer-Specific Survival of Patients Who Received Radiotherapy
eFigure 11. Breast Cancer-Specific Survival of Patients Who Received Chemotherapy

This supplementary material has been provided by the authors to give readers additional information about their work.

© 2020 Li Y et al. *JAMA Network Open*.

eTable1. Median or mean survival (months) and 10- and 20-year breast cancer-specific survival in each subtypes, SEER data source, 1990–2015.

Subgroup	ER+/PR+				ER+/PR-				ER-/PR+				ER-/PR-			
	Median survival (95%CI)	Mean survival (95%CI)	10-year BCSS (95%CI)	20-year BCSS (95%CI)	Median survival (95%CI)	Mean survival (95%CI)	10-year BCSS (95%CI)	20-year BCSS (95%CI)	Median survival (95%CI)	Mean survival (95%CI)	10-year BCSS (95%CI)	20-year BCSS (95%CI)	Median survival (95%CI)	Mean survival (95%CI)	10-year BCSS (95%CI)	20-year BCSS (95%CI)
All	NA	261.3 (260.9-261.7)	86.0% (85.8%-86.2%)	76.5% (76.3%-76.7%)	NA	241.2 (240.2-242.2)	77.9% (77.5%-78.3%)	69.8% (69.2%-70.4%)	NA	233.6 (231.3-235.9)	75.0% (74.2%-75.8%)	68.5% (67.5%-69.5%)	NA	227.6 (226.8-228.5)	72.0% (71.8%-72.2%)	67.6% (67.2%-68.0%)
Sex																
Male	NA	230.1 (224.0-236.1)	75.4% (73.6%-77.2%)	62.4% (58.5%-66.3%)	NA	188.9 (175.6-202.2)	61.0% (55.7%-66.3%)	52.9% (44.6%-59.2%)	NA	185.5 (150.1-221.0)	63.2% (47.9%-78.5%)	56.9% (38.9%-74.9%)	NA	163.3 (144.4-182.3)	58.1% (50.3%-65.9%)	50.0% (39.8%-60.2%)
Female	NA	261.5 (261.1-262.0)	86.1% (85.9%-86.3%)	76.6% (76.4%-76.8%)	NA	241.5 (240.5-242.4)	78.0% (77.6%-78.4%)	69.9% (69.3%-70.5%)	NA	233.7 (231.4-236.1)	75.0% (74.2%-75.8%)	68.6% (67.6%-69.6%)	NA	227.7 (226.9-228.4)	72.0% (71.8%-72.2%)	67.7% (67.3%-68.1%)
Race																
White	NA	262.8 (262.4-263.3)	86.5% (86.3%-86.7%)	77.1% (76.9%-77.3%)	NA	243.3 (242.2-244.4)	78.7% (78.3%-79.1%)	70.5% (69.9%-71.1%)	NA	235.7 (233.1-238.3)	75.7% (74.7%-76.7%)	69.3% (68.1%-70.5%)	NA	230 (229.5-231.2)	73.0% (72.6-73.4%)	68.5% (68.1%-68.9%)
Black	NA	234.6 (232.8-236.5)	77.2% (76.6%-77.8%)	65.4% (64.2%-66.6%)	NA	213.3 (209.7-217.0)	68.8% (67.6%-70.0%)	59.1% (56.9%-61.3%)	NA	212.3 (205.4-219.3)	67.6% (65.2%-70.0%)	60.6% (57.3%-63.9%)	NA	207.4 (205.4-209.4)	64.8% (64.2%-65.4%)	60.7% (59.7%-61.7%)

© 2020 Li Y et al. *JAMA Network Open.*

Other	NA	268.2 (266.8-269.6)	88.2% (87.8%-88.6%)	79.4% (78.6%-80.2%)	NA	251.8 (248.4-255.1)	81.1% (79.9%-82.3%)	74.7% (72.9%-76.5%)	NA	245.9 (238.7-253.2)	78.9% (76.4%-81.4%)	73.0% (69.7%-76.3%)	NA	241.1 (238.6-243.7)	77.2% (76.4%-78.0%)	72.4% (71.2%-73.6%)
Adjusted AJCC sixth edition stage																
Stage 0-II	NA	279.7 (279.3-280.1)	92.3% (92.1%-92.5%)	83.9% (83.7%-84.1%)	NA	269.7 (268.7-270.7)	88.0% (87.6%-88.4%)	80.3% (79.7%-80.9%)	NA	265.1 (262.8-267.4)	85.8% (85.0%-86.6%)	79.7% (78.5%-80.9%)	NA	259.5 (258.7-260.3)	83.1% (82.9%-83.3%)	78.5% (78.1%-78.9%)
Stage III-IV	136.0 (133.0-139.0)	165.4 (164.1-166.7)	52.8% (52.4%-53.2%)	38.2% (36.4%-39.0%)	76.0 (73.3-78.7)	136.2 (133.7-138.6)	40.7% (39.9%-41.5%)	31.1% (29.7%-32.5%)	60.0 (53.4-66.6)	130.6 (125.1-136.1)	39.5% (37.5%-41.5%)	31.4% (29.0%-33.6%)	47.0 (45.5-48.5)	131.9 (130.2-133.6)	38.8% (38.2%-39.4%)	34.8% (34.0%-35.6%)
Tumor grade																
I	NA	287.6 (286.8-288.5)	94.8% (94.6%-95.0%)	87.9% (87.3%-88.5%)	NA	279.8 (277.8-281.9)	92.1% (91.5%-92.7%)	85.7% (84.3%-87.1%)	NA	285.7 (280.3-291.2)	94.8% (93.2%-96.4%)	88.1% (85.0%-91.2%)	NA	265.7 (261.7-269.7)	86.3% (84.9%-87.7%)	80.5% (78.3%-82.7%)
II	NA	265.7 (265.0-266.3)	88.7% (88.5%-88.9%)	77.9% (77.5%-78.3%)	NA	247.6 (245.9-249.2)	80.7% (80.1%-81.3%)	71.5% (70.5%-72.5%)	NA	249.7 (245.4-254.0)	81.2% (79.6%-82.8%)	73.4% (71.2%-75.6%)	NA	235.1 (233.2-236.9)	75.2% (72.6%-75.8%)	68.9% (67.9%-69.9%)
III	NA	234.4 (233.4-234.3)	76.0% (75.6%-76.3%)	65.9% (65.3%-66.5%)	NA	222.3 (220.5-224.1)	70.6% (70.0%-71.2%)	63.4% (62.4%-64.4%)	NA	222.1 (218.7-225.5)	70.3% (69.1%-71.5%)	64.7% (63.1%-66.3%)	NA	226.2 (225.2-227.1)	71.2% (70.8%-71.6%)	67.5% (67.1%-67.9%)
IV	NA	233.1 (229.1-237.1)	75.9% (74.5%-77.3%)	65.9% (63.9%-67.9%)	NA	221.6 (213.6-229.7)	69.2% (66.3%-72.1%)	64.1% (60.6%-67.6%)	NA	220.0 (205.9-234.0)	68.4% (63.3%-73.5%)	66.5% (61.0%-72.0%)	NA	220.9 (216.7-225.0)	69.7% (68.3%-71.1%)	65.8% (64.0%-67.6%)

© 2020 Li Y et al. *JAMA Network Open*.

Histology type																
IDC 8500/3	NA	262.5 (262.0-262.9)	86.3% (86.1%-86.5%)	77.0% (76.6%-77.4%)	NA	243.3 (242.2-244.5)	78.5% (78.1%-78.9%)	70.9% (70.3%-71.5%)	NA	235.4 (232.8-238.1)	75.4% (74.4%-76.4%)	69.4% (68.2%-70.6%)	NA	230.6 (229.7-231.4)	72.9% (72.7%-73.1%)	68.7% (68.3%-69.1%)
ILC 8520/3	NA	250.0 (248.5-251.5)	83.2% (82.8%-83.6%)	70.9% (69.9%-71.9%)	NA	229.3 (226.0-232.5)	75.1% (74.1%-76.1%)	64.8% (62.8%-66.8%)	NA	238.0 (227.9-248.1)	81.0% (77.5%-84.5%)	64.6% (59.1%-70.1%)	NA	198.8 (192.8-204.9)	64.0% (61.8%-66.2%)	55.0% (52.1%-57.9%)
Mixed IDC/ILC 8522/3	NA	261.5 (260.0-263.0)	86.4% (86.0%-86.8%)	76.0% (75.0%-77.0%)	NA	244.1 (240.2-248.1)	79.8% (78.6%-81.0%)	68.9% (66.4%-71.4%)	NA	233.8 (223.2-244.5)	75.2% (71.1%-79.3%)	67.0% (61.7%-72.3%)	NA	208.8 (203.3-214.2)	65.9% (63.9%-67.9%)	60.0% (57.5%-62.5%)
Other types	NA	263.5 (262.4-264.7)	86.0% (85.6%-86.4%)	78.4% (77.6%-79.2%)	NA	235.3 (232.5-238.2)	75.9% (74.9%-76.9%)	68.3% (66.7%-69.9%)	NA	223.3 (216.9-229.7)	70.9% (68.5%-73.3%)	66.3% (63.8%-68.8%)	NA	218.1 (216.1-220.0)	69.0% (68.4%-69.6%)	64.7% (63.9%-65.5%)
ERBB2 status																
Negative	NA	68.2 (68.1-68.3)	NA	NA	NA	64.1 (63.8-64.4)	NA	NA	NA	59.6 (58.5-60.8)	NA	NA	NA	60.1 (59.8-60.4)	NA	NA
Positive	NA	67.0 (66.8-67.3)	NA	NA	NA	64.4 (63.9-64.9)	NA	NA	NA	62.7 (61.2-64.2)	NA	NA	NA	63.1 (62.8-63.5)	NA	NA
Surgery																
No	61.0 (59.2-62.8)	94.3 (92.6-95.6)	34.1% (33.1%-35.1%)	NA	36.0 (34.2-37.8)	72.7 (69.9-75.5)	24.0% (22.2%-25.8%)	NA	20.0 (16.4-23.6)	67.3 (59.1-75.5)	24.9% (19.8%-30.0%)	NA	23.0 (21.9-24.1)	72.8 (70.6-75.0)	27.7% (26.5%-28.9%)	NA

© 2020 Li Y et al. *JAMA Network Open*.

Yes	NA	195.0 (194.8-195.2)	89.0% (88.8%-89.2%)	NA	NA	183.8 (183.2-184.3)	82.4% (82.0%-82.8%)	NA	NA	176.8 (175.1-178.6)	79.1% (78.1%-80.1%)	NA	NA	171.4 (170.9-171.9)	76.0% (75.8%-76.2%)	NA
Chemotherapy	NA	246.1 (245.3-246.8)	81.2% (81.0%-81.4%)	69.3% (68.7%-69.9%)	NA	225.3 (223.6-227.0)	72.3% (71.7%-72.9%)	63.2% (62.2%-64.2%)	NA	223.9 (220.6-227.1)	71.6% (70.4%-72.8%)	64.5% (62.9%-66.1%)	NA	224.2 (223.2-225.2)	70.6% (70.2%-71.0%)	66.4% (66.0%-66.8%)
Radiotherapy	NA	269.6 (269.0-270.2)	88.8% (88.6%-89.0%)	79.8% (79.4%-80.2%)	NA	251.2 (249.8-252.6)	81.5% (81.1%-81.9%)	73.1% (72.3%-73.9%)	NA	244.8 (241.5-248.0)	78.6% (77.4%-79.8%)	72.5% (70.9%-74.1%)	NA	235.7 (234.6-236.8)	74.8% (74.4%-75.2%)	70.1% (69.5%-70.7%)

Abbreviations: ER, estrogen receptor; PR, progesterone receptor; IDC indicates invasive ductal carcinoma; ILC, invasive lobular carcinoma; NA, not available.

© 2020 Li Y et al. *JAMA Network Open*.

eTable2. Hazard ratios and Log-rank *P*-values of breast cancer-specific survival in subgroup analyses.

Subgroup	ER+/PR- vs. ER+/PR+		ER+/PR- vs. ER-/PR-		ER-/PR+ vs. ER+/PR+		ER-/PR+ vs. ER-/PR-		ER+/PR- vs. ER-/PR+		ER+/PR+ vs. ER-/PR-	
	HR (95% CI)	Log-rank *P*-value	HR (95% CI)	Log-rank *P*-value	HR (95% CI)	Log-rank *P*-value	HR (95% CI)	Log-rank *P*-value	HR (95% CI)	Log-rank *P*-value	HR (95% CI)	Log-rank *P*-value
Sex												
Male	1.81 (1.47-2.23)	<.001	0.75 (0.56-0.99)	.03	1.59 (0.85-2.94)	.07	0.75 (0.46-1.23)	.25	1.09 (0.67-1.79)	.73	0.42 (0.30-0.58)	<.001
Female	1.67 (1.63-1.71)	<.001	0.72 (0.70-0.73)	<.001	1.83 (1.75-1.92)	<.001	0.89 (0.86-0.92)	<.001	0.85 (0.82-0.88)	<.001	0.43 (0.43-0.44)	<.001
Race												
White	1.66 (1.62-1.70)	<.001	0.72 (0.71-0.74)	<.001	1.81 (1.72-1.91)	<.001	0.90 (0.86-0.93)	<.001	0.85 (0.81-0.89)	<.001	0.44 (0.43-0.45)	<.001
Black	1.54 (1.46-1.62)	<.001	0.79 (0.76-0.83)	<.001	1.58 (1.42-1.77)	<.001	0.91 (0.84-0.99)	.04	0.91 (0.84-1.01)	.06	0.53 (0.51-0.54)	<.001
Other	1.66 (1.53-1.79)	<.001	0.74 (0.69-0.80)	<.001	1.73 (1.46-2.05)	<.001	0.90 (0.79-1.03)	.13	0.86 (0.74-1.00)	.04	0.45 (0.42-0.48)	<.001
Adjusted AJCC sixth edition stage												
Stage 0-II	1.55 (1.50-1.59)	<.001	0.68 (0.66-0.70)	<.001	1.66 (1.55-1.78)	<.001	0.86 (0.81-0.90)	<.001	0.85 (0.80-0.91)	<.001	0.44 (0.43-0.45)	<.001
Stage III-IV	1.50 (1.64-1.54)	<.001	0.81 (0.79-0.83)	<.001	1.63 (1.53-1.74)	<.001	0.95 (0.91-1.00)	.06	0.89 (0.84-0.94)	<.001	0.55 (0.54-0.56)	<.001
Tumor grade												
I	1.49 (1.39-1.62)	<.001	0.58 (0.51-0.66)	<.001	1.04 (0.82-1.33)	.73	0.50 (0.41-0.61)	<.001	1.27 (1.02-1.59)	.04	0.41 (0.35-0.47)	<.001
II	1.64 (1.59-1.70)	<.001	0.72 (0.69-0.75)	<.001	1.47 (1.33-1.62)	<.001	0.75 (0.70-0.81)	<.001	1.03 (0.95-1.11)	.49	0.44 (0.43-0.46)	<.001
III	1.34 (1.30-1.38)	<.001	0.95 (0.92-0.97)	<.001	1.36 (1.29-1.43)	<.001	1.03 (0.98-1.08)	.22	0.94 (0.90-0.99)	.03	0.73 (0.71-0.74)	<.001

© 2020 Li Y et al. *JAMA Network Open*.

IV	1.26 (1.11-1.44)	<.001	0.95 (0.85-1.07)	.43	1.22 (0.98-1.52)	.05	0.99 (0.81-1.21)	.91	0.98 (0.79-1.22)	.86	0.78 (0.72-0.85)	<.001
Histology type												
IDC 8500/3	1.67 (1.63-1.71)	<.001	0.73 (0.71-0.74)	<.001	1.84 (1.74-1.95)	<.001	0.90 (0.87-0.94)	<.001	0.84 (0.80-0.88)	<.001	0.44 (0.43-0.45)	<.001
ILC 8520/3	1.56 (1.48-1.65)	<.001	0.60 (0.55-0.66)	<.001	1.23 (1.03-1.48)	.01	0.57 (0.49-0.66)	<.001	1.16 (0.98-1.36)	.09	0.40 (0.36-0.45)	<.001
Mixed IDC/ILC 8522/3	1.52 (1.41-1.64)	<.001	0.52 (0.47-0.57)	<.001	1.69 (1.35-2.11)	<.001	0.67 (0.57-0.79)	<.001	0.85 (0.70-1.03)	.07	0.34 (0.31-0.38)	<.001
Other types	1.86 (1.76-1.97)	<.001	0.72 (0.68-0.75)	<.001	2.22 (1.96-2.53)	<.001	0.93 (0.86-1.02)	.14	0.79 (0.71-0.88)	<.001	0.39 (0.37-0.40)	<.001
ERBB2 status												
Negative	2.59 (2.43-2.77)	<.001	0.61 (0.58-0.64)	<.001	4.59 (3.62-5.82)	<.001	1.05 (0.94-1.19)	.37	0.57 (0.49-0.67)	<.001	0.23 (0.22-0.26)	<.001
Positive	1.72 (1.55-1.91)	<.001	0.83 (0.76-0.90)	<.001	2.26 (1.69-3.03)	<.001	1.08 (0.88-1.33)	.46	0.76 (0.61-0.96)	.01	0.48 (0.44-0.52)	<.001
Year of diagnosis												
1990–2004	1.46 (1.42-1.49)	<.001	0.74 (0.72-0.76)	<.001	1.56 (1.48-1.64)	<.001	0.85 (0.81-0.88)	<.001	0.90 (0.86-0.94)	<.001	0.52 (0.51-0.53)	<.001
2005–2015	2.04 (1.97-2.10)	<.001	0.69 (0.68-0.71)	<.001	2.77 (2.49-3.08)	<.001	0.95 (0.89-1.01)	.10	0.73 (0.68-0.79)	<.001	0.34 (0.33-0.35)	<.001
Surgery												
No	1.48 (1.42-1.54)	<.001	0.82 (0.79-0.86)	<.001	2.00 (1.74-2.39)	<.001	1.09 (0.98-1.21)	.09	0.75 (0.66-0.84)	<.001	0.57 (0.55-0.59)	<.001
Yes	1.73 (1.68-1.78)	<.001	0.64 (0.63-0.65)	<.001	2.15 (1.99-2.31)	<.001	0.85 (0.81-0.89)	<.001	0.78 (0.73-0.83)	<.001	0.37 (0.36-0.38)	<.001
Chemotherapy	1.60 (1.56-1.65)	<.001	0.85 (0.32-0.87)	<.001	1.61 (1.52-1.70)	<.001	0.97 (0.93-1.01)	.16	0.93 (0.88-0.98)	.003	0.55 (0.54-0.56)	<.001
Radiotherapy	1.73 (1.68-1.79)	<.001	0.68 (0.66-0.70)	<.001	1.88 (1.74-2.03)	<.001	0.84 (0.80-0.89)	<.001	0.85 (0.80-0.91)	<.001	0.39 (0.38-0.40)	<.001

Abbreviations: ER, estrogen receptor; PR, progesterone receptor; IDC indicates invasive ductal carcinoma; ILC, invasive lobular carcinoma; HR, hazard radio; CI, confidence interval.

© 2020 Li Y et al. *JAMA Network Open*.

eTable3. Multivariable Cox regression analysis of breast cancer-specific survival (BCSS) and overall survival (OS)[a], SEER data source, 1990–2015.

Variable	BCSS			OS		
	Hazard Ratio	95% CI	*P*-value	Hazard Ratio	95% CI	*P*-value
Hormone receptor[b]						
ER+/PR+	0.74	0.72–0.75	<.001	0.84	0.83–0.85	<.001
ER+/PR-	1.00	Reference		1.00	Reference	
ER-/PR+	1.18	1.14–1.23	<.001	1.07	1.04–1.10	<.001
ER-/PR-	1.27	1.24–1.29	<.001	1.15	1.13–1.17	<.001
Hormone receptor[c]						
ER+/PR+	0.62	0.60–0.65	<.001	0.79	0.77–0.81	<.001
ER+/PR-	0.85	0.81–0.88	<.001	0.94	0.91–0.96	<.001
ER-/PR+	1.00	Reference		1.00	Reference	
ER-/PR-	1.07	1.03–1.11	<.001	1.08	1.05–1.11	<.001

[a]Adjusted for age at diagnosis, sex, race, tumor size, lymph node, distant metastasis, grade, and histology.
[b]ER+/PR- subtype was set as reference.
[c]ER-/PR+ subtype was set as reference.

 © 2020 Li Y et al. *JAMA Network Open*.

eFigure 1. Breast cancer-specific survival stratified by sex.

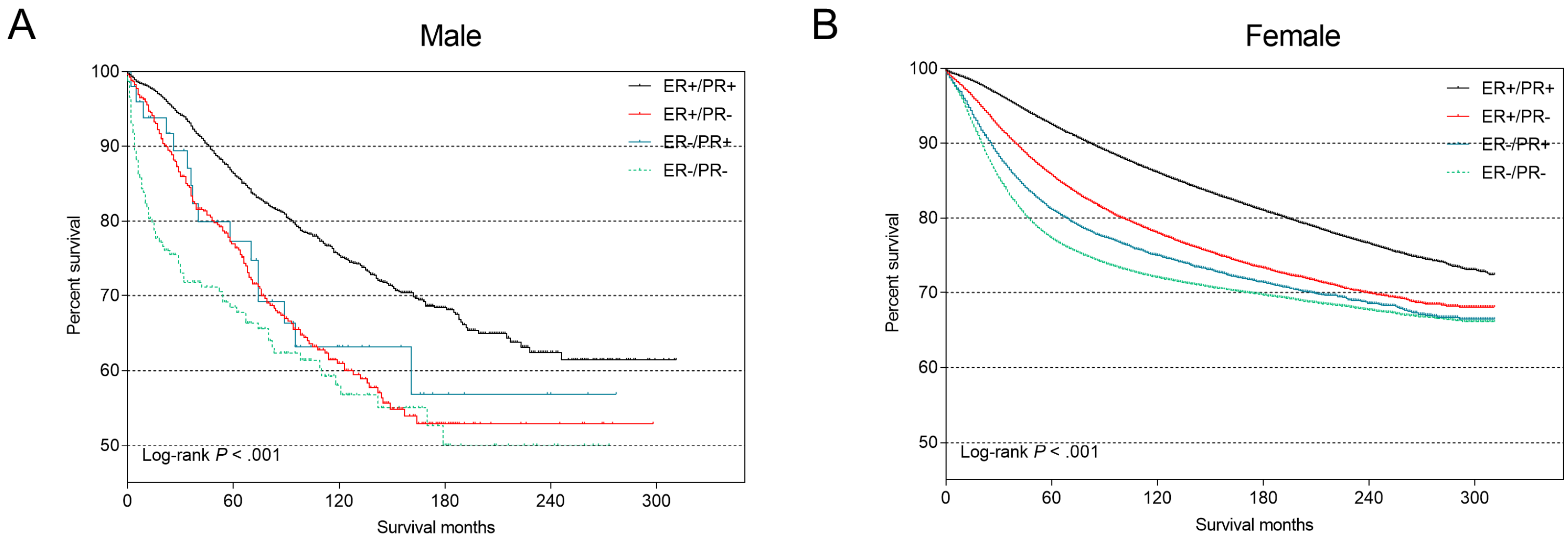

A – No. at risk

ER+/PR+	4570	2107	776	250	69	6
ER+/PR-	542	270	128	33	8	1
ER-/PR+	50	31	16	7	3	1
ER-/PR-	235	99	47	19	7	1

B – No. at risk

ER+/PR+	548595	314719	156226	58092	17319	1428
ER+/PR-	99777	55904	27849	9773	3077	260
ER-/PR+	13318	8115	5405	3007	1359	131
ER-/PR-	156312	82588	44274	17735	6302	561

Breast cancer-specific survival of breast cancer patients stratified by (A) male and (B) female.

© 2020 Li Y et al. *JAMA Network Open*.

eFigure 2. Breast cancer-specific survival stratified by race.

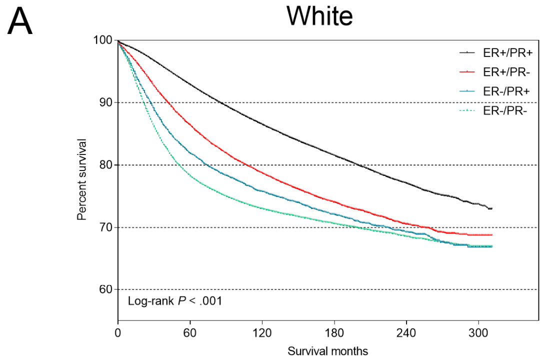

No. at risk

ER+/PR+	459856	268754	134832	50002	14977	1237
ER+/PR-	81126	46820	23690	8314	2595	230
ER-/PR+	10200	6389	4304	2383	1082	106
ER-/PR-	116920	63598	34662	14048	5035	445

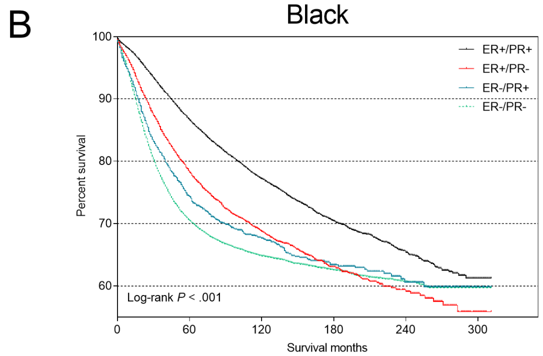

No. at risk

ER+/PR+	44235	21599	9073	3003	857	64
ER+/PR-	10953	4958	2102	653	205	17
ER-/PR+	1902	967	570	293	123	10
ER-/PR-	26281	12061	5958	2139	725	65

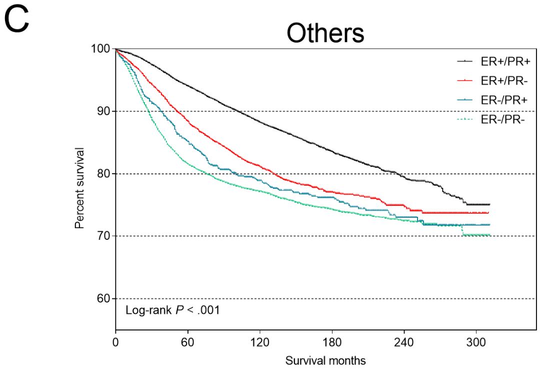

No. at risk

ER+/PR+	46182	25354	12668	5188	1516	131
ER+/PR-	7785	4209	2103	813	277	13
ER-/PR+	1206	769	530	330	154	17
ER-/PR-	12715	6761	3594	1532	541	51

Breast cancer-specific survival of breast cancer patients stratified by (A) white, (B) black, and (C) other races.

 © 2020 Li Y et al. *JAMA Network Open*.

eFigure 3. Breast cancer-specific survival stratified by AJCC stage.

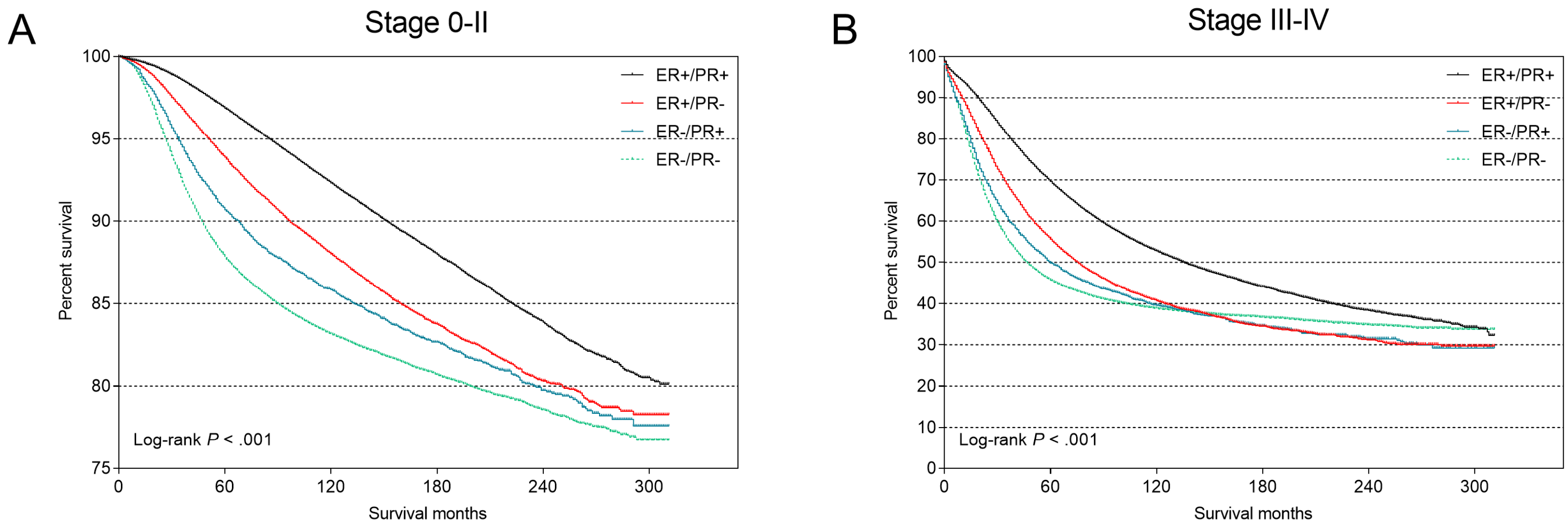

No. at risk (A)

ER+/PR+	450825	268998	135863	50335	14834	1211
ER+/PR-	75717	46565	23882	8376	2612	213
ER-/PR+	9694	6602	4486	2506	1122	99
ER-/PR-	112737	67794	36933	14685	5188	455

No. at risk (B)

ER+/PR+	80282	35077	14169	4777	1357	113
ER+/PR-	19961	7108	2759	831	263	25
ER-/PR+	2926	1084	596	291	124	15
ER-/PR-	36529	11180	5218	1927	636	50

Breast cancer-specific survival of breast cancer patients in (A) stage 0-II and (B) stage III-IV.

© 2020 Li Y et al. *JAMA Network Open*.

eFigure 4. Breast cancer-specific survival stratified by pathological grade.

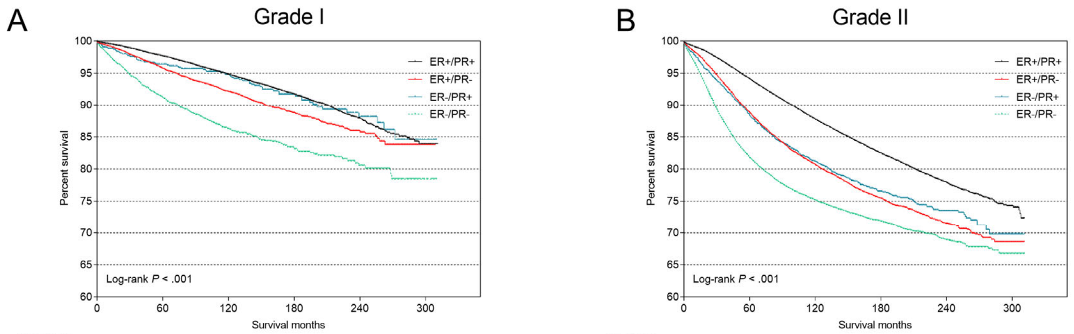

No. at risk (A, Grade I)

	0	60	120	180	240	300
ER+/PR+	135287	77952	37038	11762	2448	111
ER+/PR-	16269	10563	5503	1684	370	16
ER-/PR+	864	744	584	313	142	9
ER-/PR-	3643	2448	1439	612	208	22

No. at risk (B, Grade II)

	0	60	120	180	240	300
ER+/PR+	253316	144019	69570	24684	6447	400
ER+/PR-	38301	22277	10955	3596	949	56
ER-/PR+	3212	2295	1589	889	374	27
ER-/PR-	27608	15216	7923	3131	1053	62

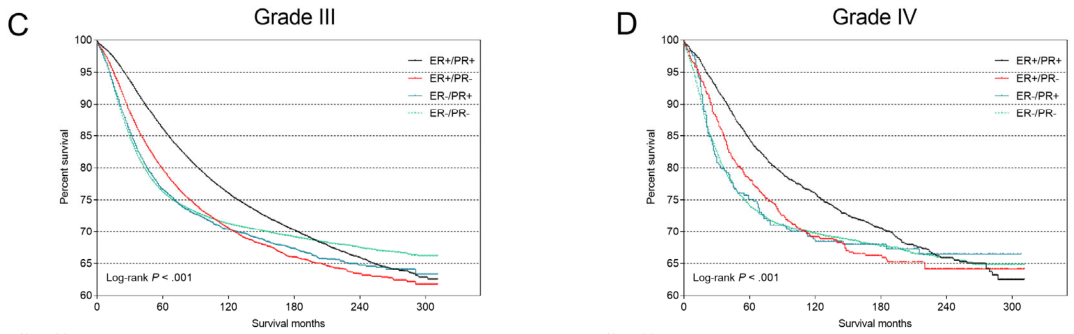

No. at risk (C, Grade III)

	0	60	120	180	240	300
ER+/PR+	117022	64040	31030	11603	3441	263
ER+/PR-	34853	16983	7759	2684	867	72
ER-/PR+	7454	3898	2342	1208	476	46
ER-/PR-	108225	55356	28689	10496	3116	234

No. at risk (D, Grade IV)

	0	60	120	180	240	300
ER+/PR+	4192	3072	2019	1058	408	32
ER+/PR-	1179	755	438	207	82	10
ER-/PR+	336	229	171	104	51	4
ER-/PR-	4406	2809	1882	937	383	19

Breast cancer-specific survival of breast cancer patients stratified by (A) grade I, (B) grade II, (C) grade III, and (D) grade IV.

© 2020 Li Y et al. *JAMA Network Open*.

eFigure 5. Breast cancer-specific survival stratified by histology.

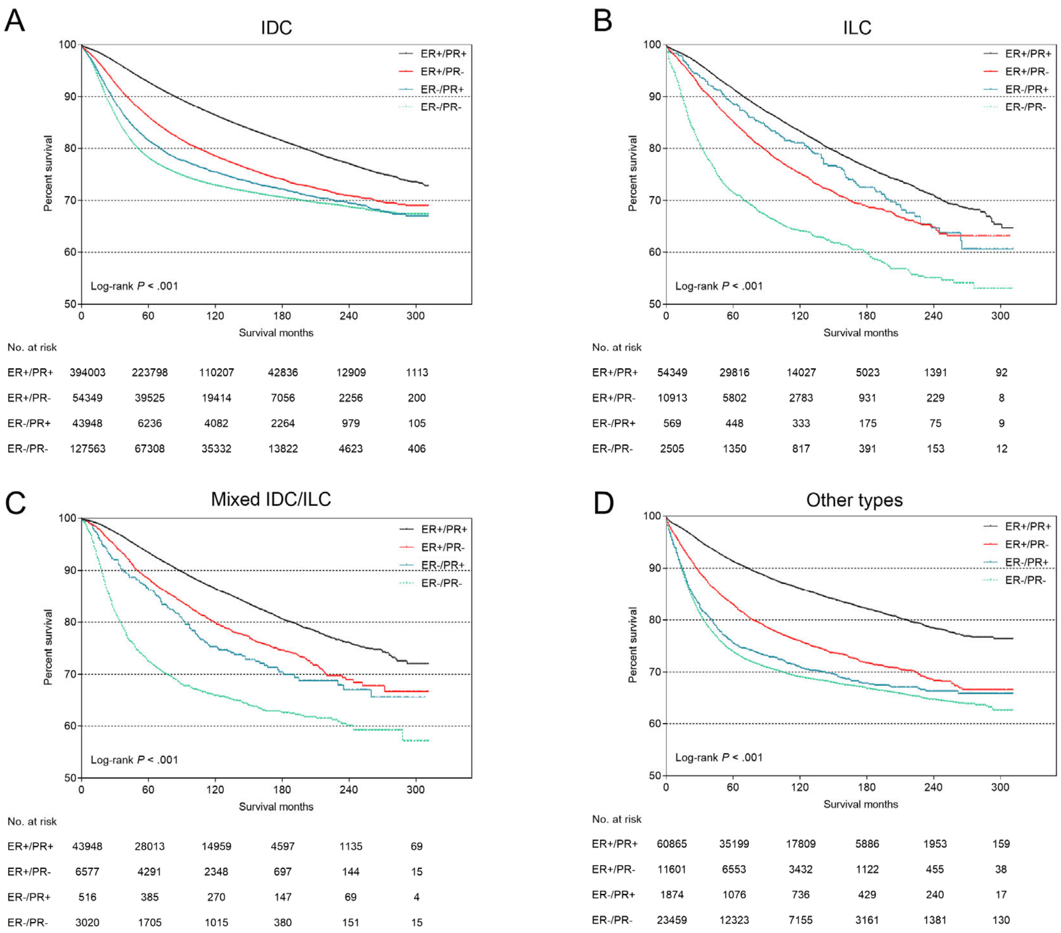

Breast cancer-specific survival of breast cancer patients stratified by (A) IDC, (B) ILC, (C) mixed IDC/ILC, and (D) other types.

© 2020 Li Y et al. *JAMA Network Open*.

eFigure 6. Breast cancer-specific survival stratified by ERBB2 status.

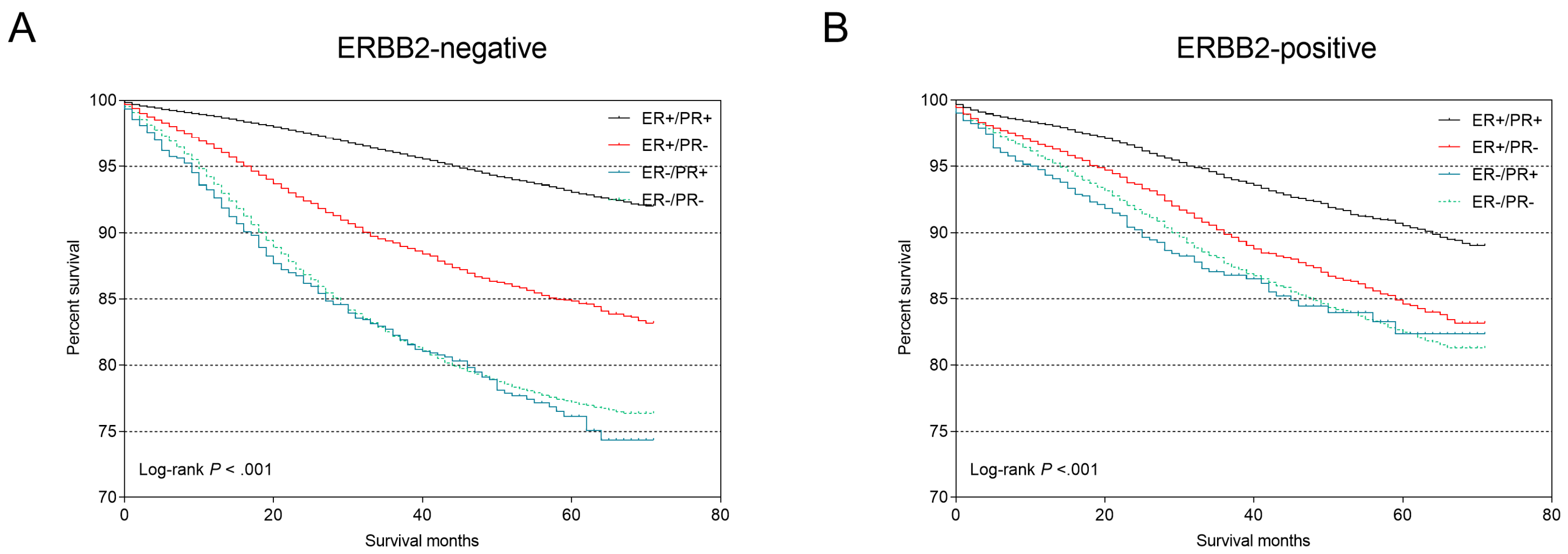

A — No. at risk

ER+/PR+	176474	115282	64416	20660
ER+/PR-	23466	14974	8466	2756
ER-/PR+	2023	1214	632	185
ER-/PR-	31883	19368	10432	3399

B — No. at risk

ER+/PR+	21669	13440	7286	2403
ER+/PR-	7943	5046	2707	882
ER-/PR+	911	574	290	86
ER-/PR-	13322	8090	4309	1386

Breast cancer-specific survival of breast cancer patients stratified by (A) ERBB2-negative and (B) ERBB2-positive.

© 2020 Li Y et al. *JAMA Network Open*.

eFigure 7. Breast cancer-specific survival of ERBB2-positive and ERBB2-negative subgroups in each hormone receptor subgroup.

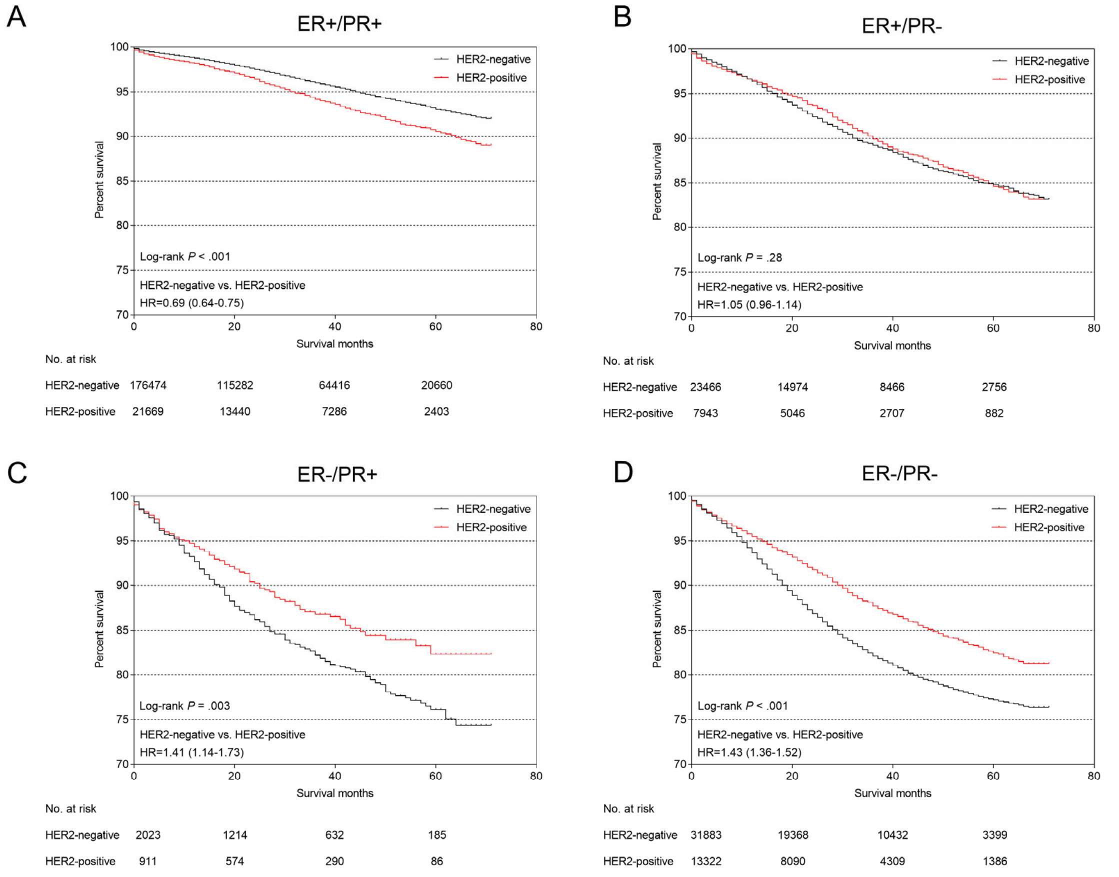

Breast cancer-specific survival of ERBB2-positive/-negative patients in (A) ER+/PR+, (B) ER+/PR-, (C) ER-/PR+, and (D) ER-/PR- subtypes.

© 2020 Li Y et al. *JAMA Network Open*.

eFigure 8. Breast cancer-specific survival stratified by year of diagnosis.

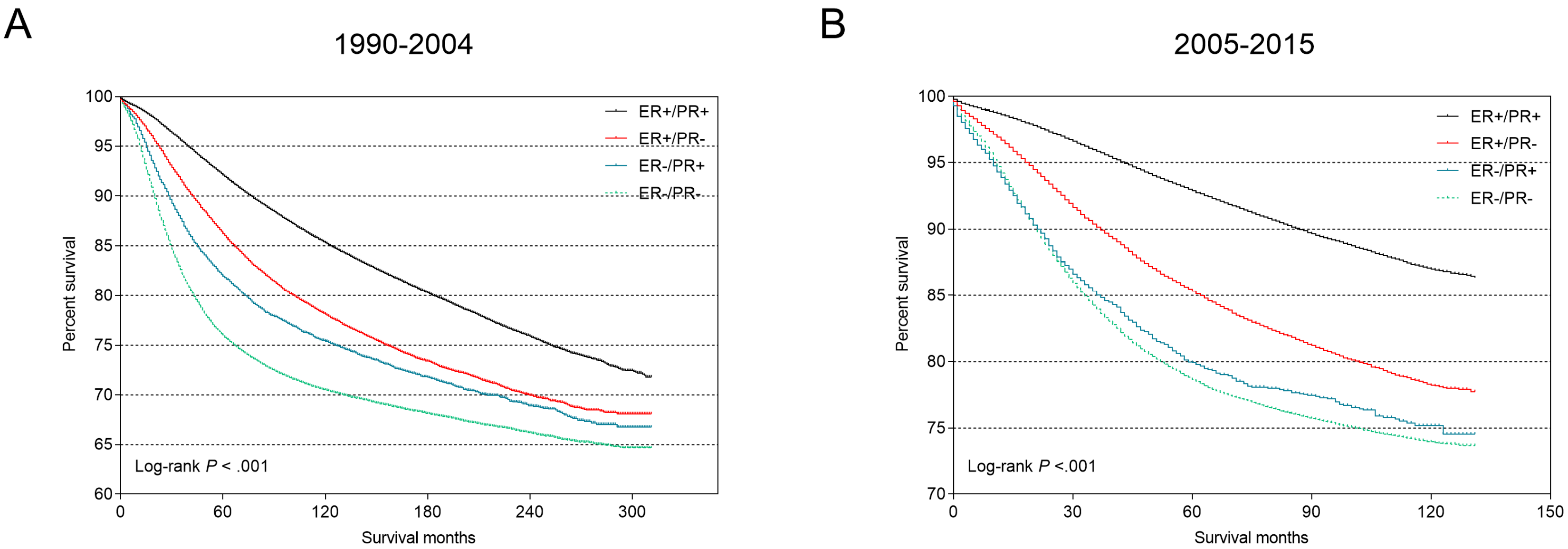

A — No. at risk

ER+/PR+	205585	173536	140694	58342	17388	1433
ER+/PR-	41342	32213	25173	9806	3084	260
ER-/PR+	7908	6092	5114	3013	1362	131
ER-/PR-	66630	47046	39505	17754	6308	561

B — No. at risk

ER+/PR+	347580	234160	143290	71225	16308
ER+/PR-	58977	39064	23958	11979	2806
ER-/PR+	5460	3392	2053	1210	307
ER-/PR-	89917	56756	35640	19237	4814

Breast cancer-specific survival of breast cancer patients who diagnosed between 1990 and 2004 (A) and between 2005 and 2015 (B).

© 2020 Li Y et al. *JAMA Network Open*.

eFigure 9. Breast cancer-specific survival stratified by patients who received surgery or not.

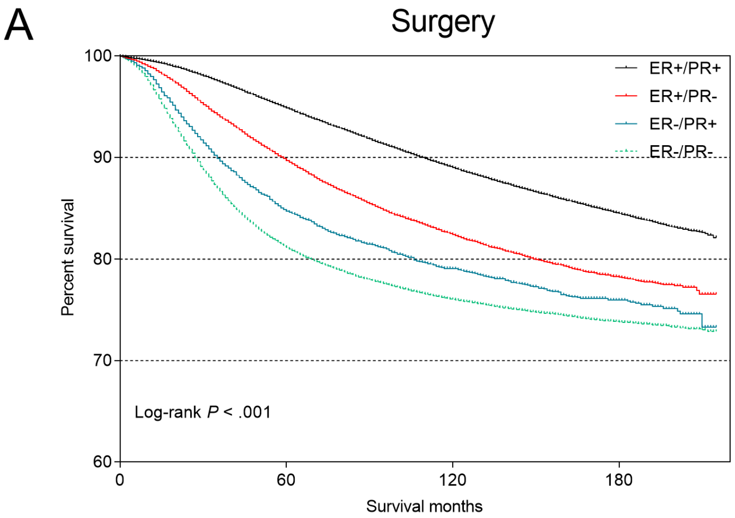

No. at risk

	0	60	120	180
ER+/PR+	459120	257118	112379	24141
ER+/PR-	79987	45092	20081	3920
ER-/PR+	8581	4969	2803	787
ER-/PR-	123828	65945	31062	6754

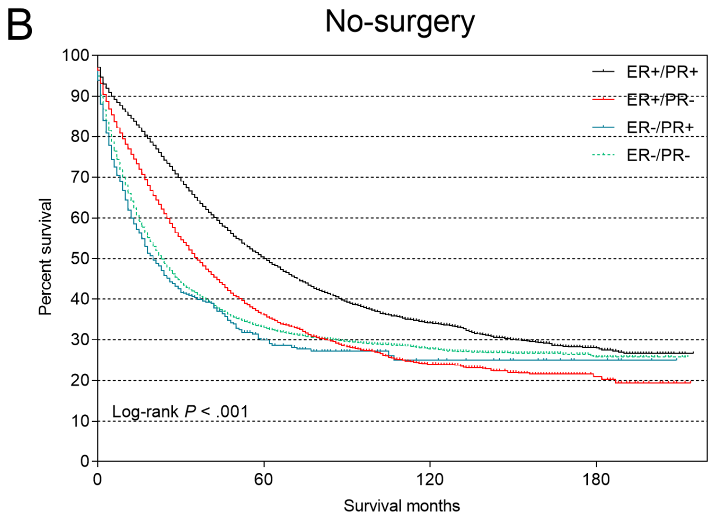

No. at risk

	0	60	120	180
ER+/PR+	27094	4895	1079	148
ER+/PR-	6870	1021	219	33
ER-/PR+	734	76	22	6
ER-/PR-	10693	1458	432	72

Breast cancer-specific survival of breast cancer patients who received surgery (A) or (B) not.

© 2020 Li Y et al. *JAMA Network Open*.

eFigure 10. Breast cancer-specific survival of patients who received radiotherapy.

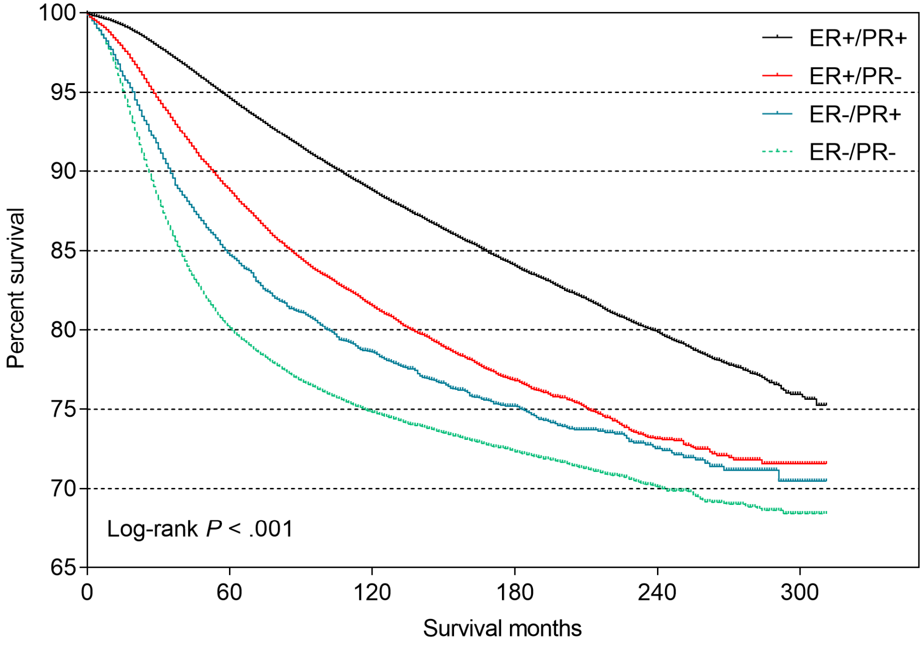

No. at risk

ER+/PR+	283848	171704	87374	31621	8323	535
ER+/PR-	48389	29308	14994	4985	1353	90
ER-/PR+	6134	3984	2666	1423	602	61
ER-/PR-	73065	41559	22836	8627	2654	190

© 2020 Li Y et al. *JAMA Network Open*.

eFigure 11. Breast cancer-specific survival of patients who received chemotherapy.

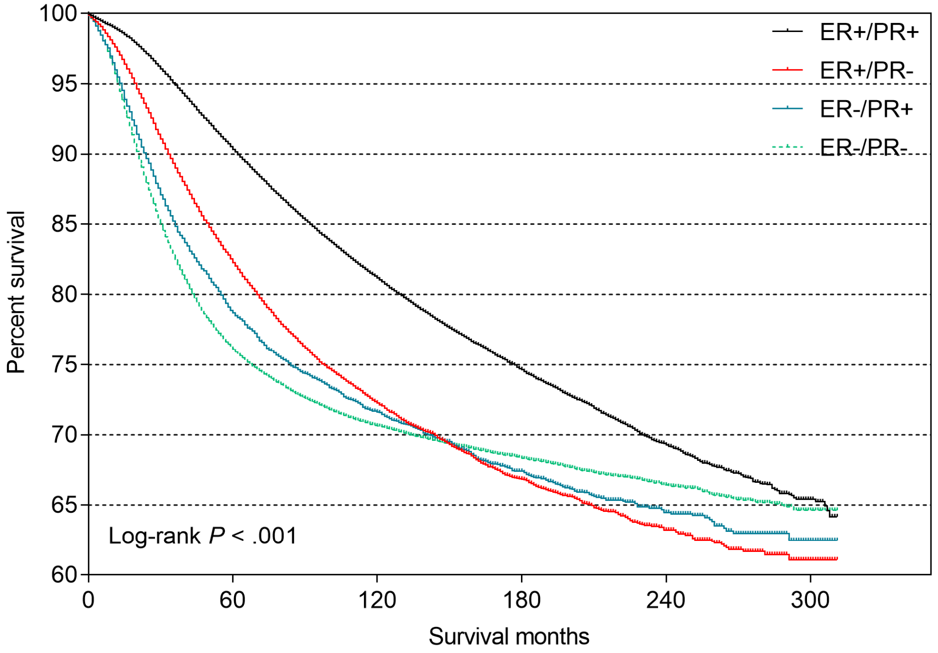

No. at risk

ER+/PR+	185752	111407	55583	18836	4885	466
ER+/PR-	42416	22092	10240	3214	926	85
ER-/PR+	7754	4401	2784	1471	638	68
ER-/PR-	103493	53006	27657	10425	3380	293

© 2020 Li Y et al. *JAMA Network Open*.
